# Estimation of the Timing and Intensity of Reemergence of Respiratory Syncytial Virus Following the COVID-19 Pandemic in the US

**DOI:** 10.1001/jamanetworkopen.2021.41779

**Published:** 2021-12-16

**Authors:** Zhe Zheng, Virginia E. Pitzer, Eugene D. Shapiro, Louis J. Bont, Daniel M. Weinberger

**Affiliations:** 1 Public Health Modeling Unit, Department of Epidemiology of Microbial Diseases, Yale School of Public Health, New Haven, Connecticut; 2 Department of Pediatrics, Yale University School of Medicine, New Haven, Connecticut; 3 Department of Pediatrics, Department of Immunology, University Medical Center Utrecht, Utrecht University, Utrecht, the Netherlands; 4 ReSViNET Foundation, Zeist, the Netherlands

## Abstract

**Importance:**

Respiratory syncytial virus (RSV) is a leading cause of hospitalizations in young children. RSV largely disappeared in 2020 owing to precautions taken because of the COVID-19 pandemic. Estimating the timing and intensity of the reemergence of RSV and the age groups affected is crucial for planning for the administration of prophylactic antibodies and anticipating hospital capacity.

**Objective:**

To examine the association of different factors, including mitigation strategies, duration of maternal-derived immunity, and importation of external infections, with the dynamics of reemergent RSV epidemics.

**Design, Setting, and Participants:**

This simulation modeling study used mathematical models to reproduce the annual epidemics of RSV before the COVID-19 pandemic in New York and California. These models were modified to project the trajectory of RSV epidemics from 2020 to 2025 under different scenarios with varying stringency of mitigation measures for SARS-CoV-2. Simulations also evaluated factors likely to affect the reemergence of RSV epidemics, including introduction of the virus from out-of-state sources and decreased transplacentally acquired immunity in infants. Models using parameters fitted to similar inpatient data sets from Colorado and Florida were used to illustrate these associations in populations with biennial RSV epidemics and year-round RSV circulation, respectively. Statistical analysis was performed from February to October 2021.

**Main Outcomes and Measures:**

The primary outcome of this study was defined as the estimated number of RSV hospitalizations each month in the entire population. Secondary outcomes included the age distribution of hospitalizations among children less than 5 years of age, incidence of any RSV infection, and incidence of RSV lower respiratory tract infection.

**Results:**

Among a simulated population of 19.45 million people, virus introduction from external sources was associated with the emergence of the spring and summer epidemic in 2021. There was a tradeoff between the intensity of the spring and summer epidemic in 2021 and the intensity of the epidemic in the subsequent winter. Among children 1 year of age, the estimated incidence of RSV hospitalizations was 707 per 100 000 children per year in the 2021 and 2022 RSV season, compared with 355 per 100 000 children per year in a typical RSV season.

**Conclusions and Relevance:**

This simulation modeling study found that virus introduction from external sources was associated with the spring and summer epidemics in 2021. These findings suggest that pediatric departments should be alert to large RSV outbreaks in the coming seasons, the intensity of which could depend on the size of the spring and summer epidemic in that location. Enhanced surveillance is recommended for both prophylaxis administration and hospital capacity management.

## Key Points

**Question:**

What are the factors associated with the timing and intensity of reemergent respiratory syncytial virus (RSV) epidemics following the COVID-19 pandemic?

**Findings:**

In this simulation modeling study of a simulated population of 19.45 million people, virus introduction from external sources was associated with the spring and summer epidemics in 2021. Reemergent RSV epidemics in 2021 and 2022 were projected to be more intense and to affect patients in a broader age range than in typical RSV seasons.

**Meaning:**

These findings suggest that the timing and intensity of reemergent RSV epidemics might be different from the usual RSV season, depending on the duration of mitigation measures and the extent of virus introduction from other regions.

## Teaser

This simulation modeling study examines the association of mitigation strategies, duration of maternal-derived immunity, and importation of external infections with reemergent respiratory syncytial virus epidemics following the COVID-19 pandemic in the US.

## Introduction

Respiratory syncytial virus (RSV) infection is a leading cause of acute respiratory hospitalizations in infants, young children, and older people.^1,2,3^ Individuals develop only partial immunity following RSV infections, and this incomplete immunity permits reinfections throughout life.^4^ Although most infants are born with protective immunity against RSV infections due to antibodies from the mother that are acquired transplacentally, this protection wanes quickly.^5,6,7^

RSV epidemics follow notable spatiotemporal patterns in the United States, with consistent seasonal timing and duration.^8,9^ However, the incidence of RSV in many regions in the United States has declined since the introduction of mitigation measures for the COVID-19 pandemic in March 2020, the intensity and duration of which varied by state.^10^ The low positivity rate continued throughout the typical RSV season in the fall and winter of 2020 to 2021. Many other countries reported similarly low frequencies of RSV detection in the 2020 season.^11,12,13^

As mitigation measures have been gradually lifted, different patterns of RSV epidemics emerged in different regions in early 2021.^10,11,12,13^ Small spring and summer waves of RSV activity were reported in France, Spain, and in many US states.^10,12,14^ Large out-of-season surges of RSV infections were reported in Australia, South Africa, and in several southern US states.^11,15^ Nonetheless, even with many restrictions lifted, RSV activity remains low in many countries.^16,17^ What causes these differences remains unclear.

Anticipating the intensity, timing, and age distribution of RSV epidemics in the coming years is needed for planning for the administration of RSV prophylaxis and for projecting hospital utilization. However, many factors may affect when and to what extent RSV epidemics resume. For example, introduction of RSV from other regions may accelerate the process, and low immunity in the population due to a low incidence of reinfections may produce a large population of susceptible individuals sufficient to trigger an outbreak.

Our study aims to use simulation models to explore a range of potential scenarios and to better understand the factors associated with RSV epidemics. In this study, we used historical RSV inpatient data, combined with validated transmission dynamic models, to evaluate the potential patterns of reemergent RSV epidemics under the influence of various factors, including mitigation strategies, duration of maternal-derived immunity, and importation of external infections. We also examined how the age distribution of RSV infections and hospitalizations might be expected to change in the coming years under the most likely scenario.

## Methods

### Data

RSV-specific hospitalization data for New York (2005-2014) and California (2003-2011) were obtained from the State Inpatient Databases of the Healthcare Cost and Utilization Project maintained by the Agency for Healthcare Research and Quality.^18^ These comprehensive databases contain all hospital discharge records from community hospitals in participating states. Data sets included the month of hospitalization and the age of the patient. A hospitalization was defined as due to RSV if any of the discharge diagnostic codes included 079.6 (RSV), 466.11 (bronchiolitis due to RSV), or 480.1 (pneumonia due to RSV), based on the *International Classification of Diseases, Ninth Revision (ICD-9)*.^19^ This code set has high sensitivity and specificity.^20,21^ Information about population size in each age group was obtained from the US Census Bureau’s American Community Survey.^22^ Birth rate by year and state was obtained from the Centers for Disease Control and Prevention vital statistics.^23^ RSV surveillance data for California (2009-2018) were obtained from the Immunization Branch, California Department of Public Health and were used to validate model estimations.^24^ We also performed analyses using parameters fitted to similar inpatient data sets from Colorado and Florida to illustrate potential associations with mitigation measures in populations with biennial RSV epidemics and year-round RSV circulation, respectively.^25,26^ The analysis of the data was approved by the Human Investigation Committees at Yale University and followed the guidance for the Conduct and Reporting of Modeling and Simulation Studies in the Context of Health Technology Assessment.^27^

### Outcome Measures

The primary clinical outcome of interest was the estimated monthly number of RSV hospitalizations. Secondary outcomes of interest included the age distribution of hospitalizations among children under 5 years of age, incidence of any RSV infection, and incidence of RSV lower respiratory infection. Percentage changes in incidence were calculated by comparing the differences between the estimated incidence in the 2021 to 2022 RSV season with and without changes to the transmission rate related to mitigation measures and virus introduction from external sources (eTable 1 in the Supplement).

### Statistical Analysis

To estimate RSV transmission dynamics, we extended a previously published RSV transmission model (eAppendix, eFigure 1, eTable 1, eTable 2, eFigure 2, and eFigure 3 in the Supplement).^25^ We generated forward simulations using varying input parameters (eTable 1 and eTable 2 in the Supplement), assuming the same immigration and emigration, death rate, and birth rate as 2019. We evaluated various durations of the mitigation periods and baseline levels of imported infections.

We simulated the monthly number of RSV hospitalizations from 2021 to 2025. With many unknowns, our aim was to explore a wide range of possible scenarios and examine which factors may be associated with RSV epidemics (Table) rather than making precise forecasts. The models were initially developed prior to the RSV epidemic in spring and summer 2021 and were subsequently expanded to explore additional parameter values.

**Table.** Description of the 5 Simulations

Scenarios	Description
Stringency of mitigation measures	
1. Constant low level of transmission	Individuals obey social distancing strictly and have constant low contact rate from March 2020 to March 2021
2. Sudden decrease + gradual increase in transmission	Mitigation measures are most strict at the beginning and are gradually relaxed between March 2020 and March 2021
3. Decrease in nonhousehold contacts	Stay-at-home orders from late March 2020 to late June 2020 reduced contact opportunities in nonhousehold settings by 82% and increased household contacts by 10%
Factors associated with the reemergence of RSV epidemics	
4. Importation of external infections	Introduction of the virus from other regions may ignite RSV epidemics
5. Decrease in the duration of protective maternal immunity	Absence of RSV epidemics leads to lack of boosting of maternal immunity

Abbreviation: RSV, respiratory syncytial virus.

#### Decrease in Transmission

We evaluated a range of reductions in the RSV transmission rate, from 10% to 25%, based on the results of a previous study analyzing the impact of mitigation measures on seasonal respiratory viruses.^28^ We evaluated 2 kinds of reduction in transmission beginning in March 2020 and lasting until April 2021: a constant low level of transmission (scenario 1), and a large decrease followed by a linear increase in transmission (scenario 2). The rationale for the second type of decrease comes from the observation that mitigation measures were strictest at the beginning of the COVID-19 pandemic and were gradually relaxed with time.^29,30,31^

#### Changes in Contact Patterns

We also explored the association of heterogeneous changes in contact patterns with RSV epidemics. Specifically, we examined the outcomes of a 3-month stay-at-home order from April 1 to July 1, 2020 (scenario 3).^29,31^ We calculated the percentage of household contacts in detailed age groups and multiplied by the age-specific contact rates of respiratory-spread infectious agents in the corresponding age groups.^32,33,34^ We assumed an 82% decrease in nonhousehold contacts^35^ and a 10% increase in household contacts.^36^

#### Virus Introduction From External Sources

Stay-at-home orders and travel restrictions may also be associated with the introduction of RSV into a population. Travel restrictions between countries and states may limit the introduction of external RSV infections, whereas constant virus seeding from other regions may accelerate the reemergence of RSV epidemics. There is little data on the rate of infections with RSV that are imported from other regions. Therefore, we explored a range of 5 to 30 imported infections per 100 000 population per month (scenario 4). For this scenario, we assumed the RSV transmission rate exhibited a large decrease followed by a linear increase back to prepandemic levels (similar to scenario 2). We assumed imported infections are mild and do not lead to lower respiratory infection (LRI) or hospitalization. In the main analysis, we assumed the number of external infections dropped to 0 in April 2020 because of the implementation of travel restrictions. We assumed an absence of external infections until February 2021 and a linear increase in virus introduction back to prepandemic levels by May 2021 based on RSV activities in other states.^37^ We also explored various other scenarios of virus introduction (eFigure 4 in the Supplement).^38^

#### Decreased Duration of Transplacentally Acquired Immunity in Infants

Reduction in transmission results in low virus activity in the community, which may cause fewer exposure opportunities for the general population. Because virus exposure can boost immunity levels,^39^ pregnant women may have lower concentrations of antibodies. Correspondingly, the level and duration of the immunity in infants acquired from their mothers may also decrease. Because few previous studies quantified how virus exposure in pregnant women may affect the duration of transplacentally acquired immunity in infants, we probed the outcome of a lack of boosting by proposing a range of possible decreases in the duration of transplacentally acquired immunity on top of the linear change in RSV transmission (scenario 5). The period of shortened transplacentally acquired immunity is therefore dependent on the RSV epidemics from November 2020 to October 2021, based on observations and the results of simulations.

We initially evaluated all 5 of the aforementioned scenarios and developed the models prior to the epidemic in summer 2021. Scenario 4, in which we allowed for imported infections from external sources, best captured the observed dynamics that emerged during the spring and summer of 2021. Therefore, we focused on scenario 4 in the results. Statistical analysis was performed using R software version 4.0.5 (R Project for Statistical Computing) from February to October 2021.

## Results

**Figure 1.**
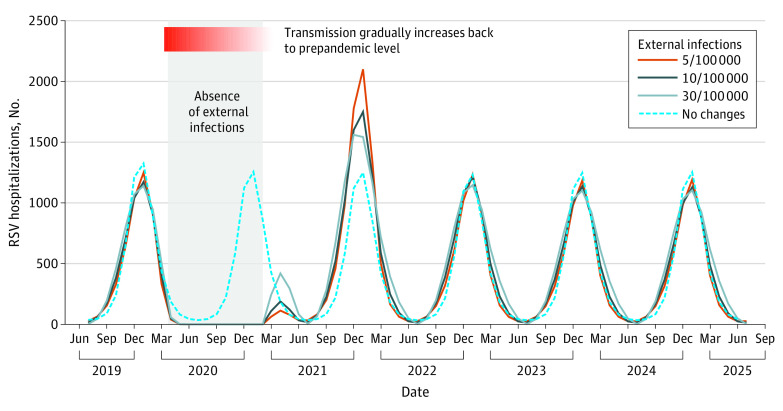
Association of Importation of External Infections and Respiratory Syncytial Virus (RSV) Hospitalizations in New York, 2019–2025 The dotted blue line corresponds to the counterfactual scenario that seasonal RSV epidemics were not interrupted. The solid lines represent a range of background importations of external infections. The gray shaded area indicates absence of external infections related to travel restrictions beginning in April 2020 and the absence of RSV activity in other regions of the US until February 2021. We assumed the viral importation rate increased linearly back to prepandemic levels over 3 months. In this scenario, we assumed RSV transmission within the population dropped to 80% of prepandemic levels in March 2020 and gradually increased back to prepandemic levels over a 13-month period.

This study included a simulated population of 19.45 million people. With virus introduction from external sources (scenario 4), an increase in RSV hospitalizations was expected in the spring and summer of 2021 (Figure 1). The intensity of the spring and summer epidemics depended on the amount of seeding; higher rates of introductions of the virus from other locations was associated with more intense spring and summer epidemics. These spring and summer epidemics were expected to be followed by earlier than usual winter epidemics. There was a tradeoff between the intensity of the spring and summer epidemic and the intensity of the subsequent winter epidemic, with larger spring and summer epidemics followed by smaller winter epidemics. Although the expected epidemic in spring and summer 2021 in New York was small (peak incidence was 419 hospitalizations per 100 000 people in April), states with less seasonal variability (eg, Florida) were expected to have had a larger summer epidemic, which was more intense than RSV epidemics in typical seasons (eFigure 5 in the Supplement). Under this scenario, epidemics were expected to revert to typical timing and intensity in the following year (Figure 1).

### Shifts in the Age Distribution of Infection During the Reemergence of RSV

**Figure 2.**
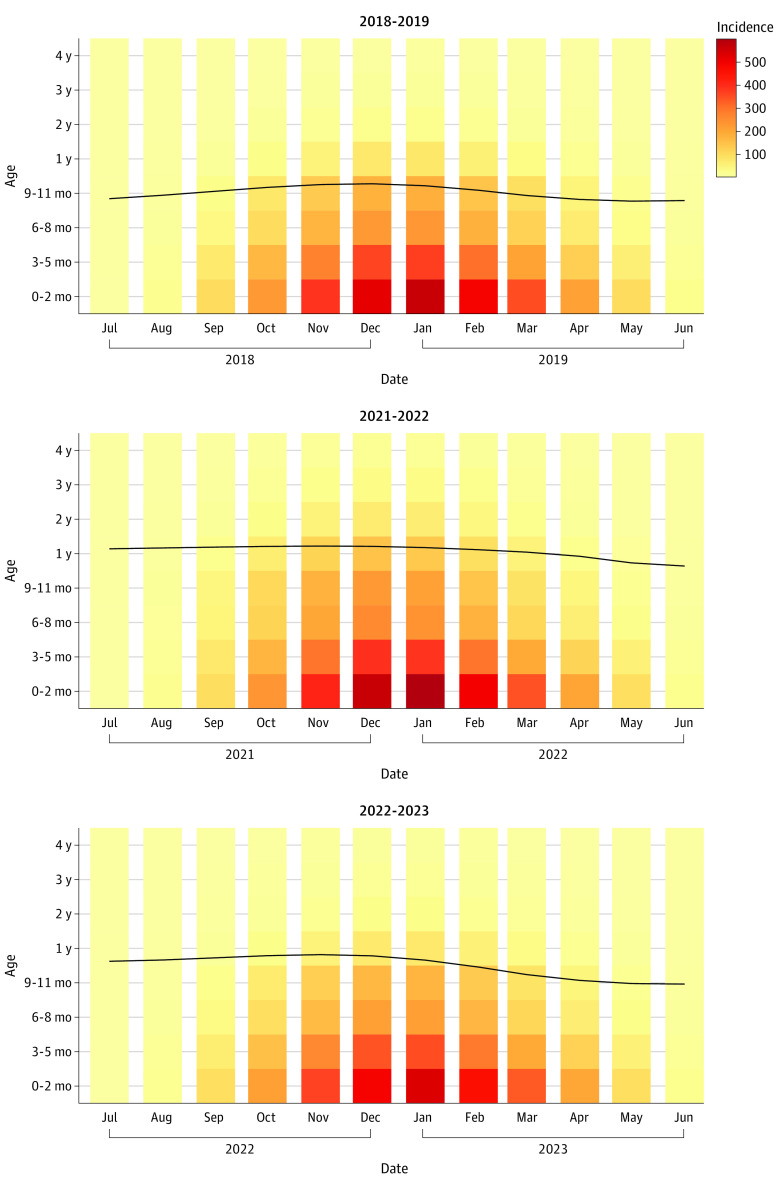
The Mean Age of RSV Hospitalizations Among Children Aged Younger Than 5 Years The background color represents the incidence of RSV hospitalization per 100 000 people per month in each age group in each month. Darker red colors indicate a higher incidence. The black line and values indicate the mean age of hospitalization (in years) varies with time.

Under scenario 4, which most closely resembled what happened in the US in terms of relaxation of pandemic measures and the emergence of a spring and summer epidemic,^29,30,31^ the mean age of hospitalization among children under 5 years was expected to be 1.17 years of age in January 2022 compared with 0.84 years of age in January 2019 (Figure 2). The mean age of hospitalization was estimated to gradually return back to the prepandemic level in 2023. Young children were expected to have higher incidence of hospitalizations during the first epidemic year following the restrictions (Figure 2). These age shifts were generally consistent across scenarios although the exact numbers were different.

**Figure 3.**
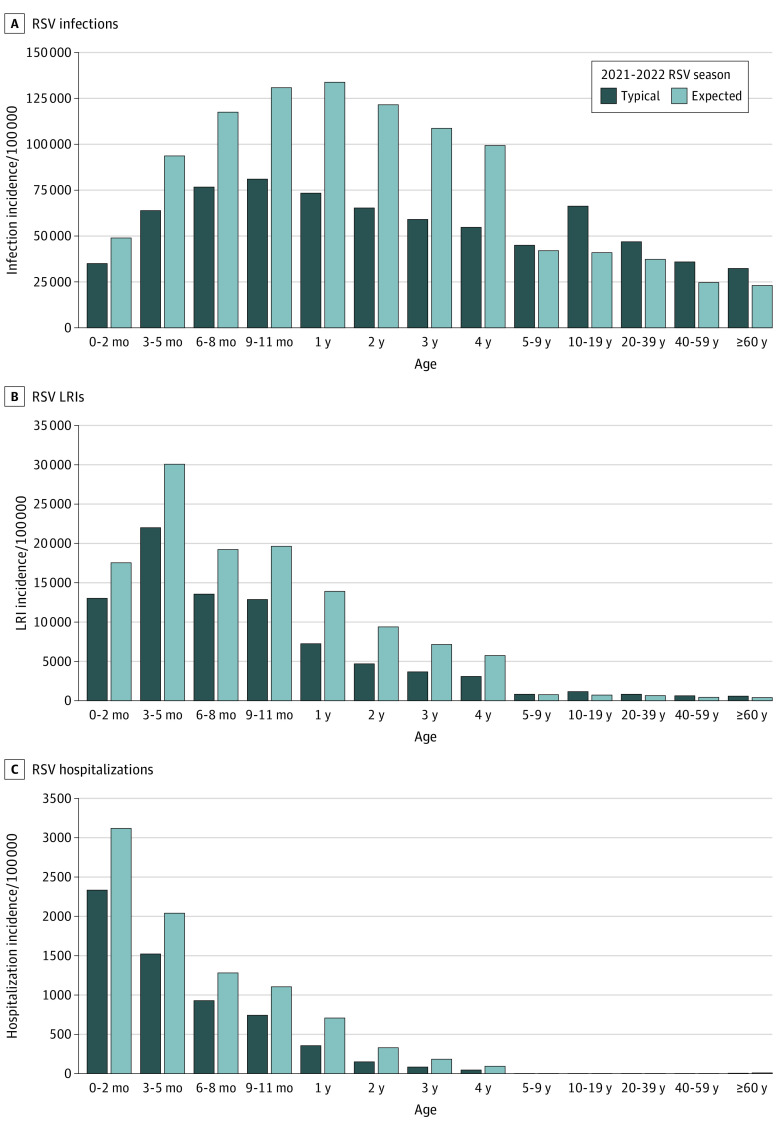
Age Distribution of Respiratory Syncytial Virus (RSV) Infections, Lower Respiratory Infections (LRIs), and Hospitalizations, 2021-2022 RSV Season The figures show a comparison of RSV infections (A), RSV LRIs (B), and RSV hospitalizations (C) during a typical season (counterfactual incidence of RSV cases during the 2021-2022 RSV season if there was no COVID-19 pandemic and no mitigation measures in place) and the expected season (expected incidence of RSV cases during the 2021-2022 RSV season under the assumption that substantial virus importation was disrupted by mitigation measures between April 2020 and February 2021).

Across all age groups, children aged 1 to 4 years were expected to have the greatest relative increase in the incidence of RSV infection (82%-86%), LRI (87%-101%) and hospitalization (99%-119%) compared with a typical prepandemic RSV season (Figure 3). Among children 1 year of age, the estimated incidence of RSV hospitalizations was 707 per 100 000 children per year in the 2021 and 2022 RSV season, compared with 355 per 100 000 children per year in a typical RSV season. However, infants aged 3 to 5 months were expected to continue to have the largest absolute incidence of LRI (30 075 LRIs per 100 000 infants per year), and infants younger than 3 months were expected to continue to have the largest incidence of RSV hospitalization (3116 hospitalizations per 100 000 infants per year). Without virus importation, the risk of RSV infections across all age groups in the winter of 2021 and 2022 would be greater, as more susceptible individuals were spared from infections in the absence of summer epidemics (eFigure 6 in the Supplement).

### Association of the Stringency of Mitigation Measures and Duration of Protective Immunity in Infants With Epidemic Timing

**Figure 4.**
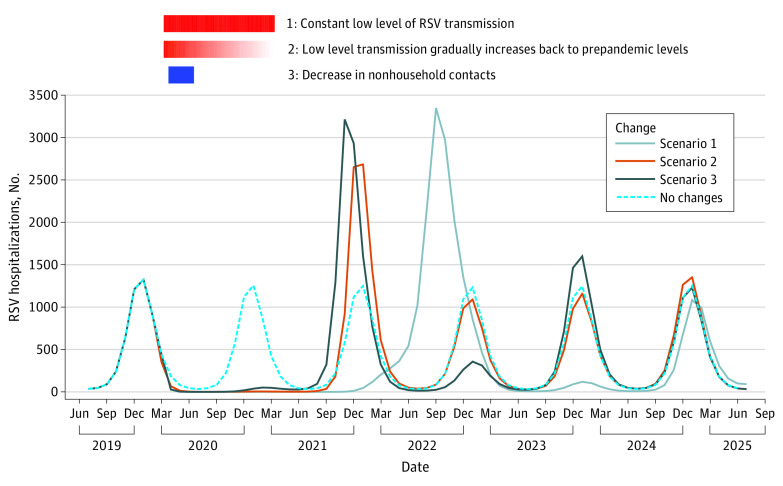
The Association of Mitigation Measures and RSV Hospitalizations Without External Infections, New York, 2019–2025 All scenarios in this figure assumed virus circulated locally without external sources of viral importation, both before pandemic and during pandemic. The dotted blue line shows the counterfactual scenario that there is no COVID-19 pandemic and no mitigation measures in place. The solid lines show 3 scenarios of stringency of mitigation measures. Scenario 1 was characterized by constant low level of transmission (80% prepandemic level) from March 2020 to March 2021. Scenario 2 was characterized by sudden 20% decrease in RSV transmission in March 2020 followed by a linear increase back to prepandemic levels. Scenario 3 was characterized by an 82% decrease in nonhousehold contacts and 10% increase in household contacts between April 1 and July 1, 2020. The solid red rectangle on the top indicates a constant strict mitigation measure lasting for 13 months, from March 2020 to March 2021. The gradient red rectangle in the middle indicates a gradually relaxed mitigation measure that lasts for the same period of time (March 2020 to March 2021). The blue rectangle on the bottom indicates a 3-month stay-at-home order starting at the end of March 2020.

The scenarios that assumed no external introduction of RSV infections all failed to generate notable summer epidemics in 2021. Without imported infections, the timing of reemergent RSV epidemics depended on the stringency of mitigation measures. If we assumed a long-lasting low level of RSV transmission (scenario 1), it took more than a year for RSV activity to resume with an out-of-season outbreak in the summer of 2022 (peak incidence was 3348 hospitalizations per 100 000 people in September 2022) (Figure 4). In contrast, when RSV transmission gradually increased back to prepandemic levels (scenario 2), a large outbreak was estimated to occur in the winter of 2021 and 2022 (peak incidence was 2684 hospitalizations per 100 000 people in January 2022) (Figure 4). Models with smaller decreases in transmission generally generated earlier reemergence of RSV epidemics (eFigure 7 in the Supplement). With the short and strict stay-at-home orders (scenario 3), the RSV epidemic in the 2021 and 2022 fall and winter season was estimated to have an earlier onset and peak timing than the usual RSV season (peak incidence was 3213 hospitalizations per 100 000 people in November 2021) (Figure 4). Shorter transplacentally acquired immunity in infants (scenario 5) did not substantially change the timing or the amplitude of RSV epidemics on top of the changes resulting from reduced transmission (eFigure 8 in the Supplement).

## Discussion

Seasonal RSV epidemics were disrupted by COVID-19 mitigation measures during 2020-2021. Understanding the association of different factors with the timing, intensity, and age distribution of reemergent RSV epidemics is crucial for clinical and public health decision-making. Our results suggest that the rate of importation of infections into a population was associated with the dynamics over the coming seasons. There was a tradeoff between the intensity of a spring and summer epidemic and the intensity of the epidemic the following winter. Nevertheless, RSV hospitalizations in children aged 1 to 4 years were expected to double compared with a typical RSV season during the 2021 to 2022 season even with a prominent summer epidemic.

Variations in the patterns of reemergent RSV epidemics across regions and countries could potentially be explained with our simulated results:

1. In New York, which has strong connections to other parts of the US and the world, substantial risk of virus importation likely existed prepandemic. The risk of virus importation depends both on the volume of travel and the level of external RSV activity. Before February 2021, most states in the US reported no RSV epidemics. Virus importation from other states was therefore highly unlikely. Following an increase in RSV activity in Florida, the chance of virus importation from other states increased, which may explain the small spring/summer epidemic in 2021 in New York (see Figure 1).
2. In Florida, a state that has year-round RSV transmission and weak seasonal variability, COVID-19 mitigation measures may have temporarily suppressed local RSV transmission. At the same time, travel restrictions may have substantially decreased imported infections, as Florida used to be a top destination for winter vacations in the US. After reopening, the accumulation of susceptibility and reintroduction of external infections may have triggered the large out-of-season outbreak in spring/summer 2021 (eFigure 5 in the Supplement).
3. In Argentina and Canada, governments implemented very strict mitigation measures at the start of the COVID-19 pandemic.^40,41^ Some restrictions have been extended and remained in place as of April 2021 to protect against new variants.^42,43^ It is worth noting that these 2 countries represent both the northern and southern hemisphere and have opposite RSV seasons.^44^ Nonetheless, both countries reported low RSV activity as of June 2021.

Our study builds upon a previous modeling study^28^ by incorporating age structure and evaluating the effects of external introduction of infections. Our analyses successfully captured the epidemics of RSV in spring/summer 2021 by accounting for importation of infections from external sources. More importantly, our results suggested that RSV epidemics were likely to resume annual seasonal cycles in the winter of 2021-2022, much earlier than the projections assuming no external source of infections. This updated information is valuable for planning the administration of prophylaxis and anticipating hospital capacity, as well as the resumption of clinical trials for various RSV prevention strategies.

Our research suggested that the timing and intensity of reemergent RSV epidemics was associated with virus introduction. Recent research found that the out-of-season RSV epidemics that occurred in Australia in 2020 and 2021 were clonal, suggesting a single viral introduction from external sources.^45^ A recent finding from Kenya also reported that seasonal RSV was reintroduced into communities every year from other parts of the country.^46^ Hence, high-density sampling and sequencing of RSV across a region will be critical to understanding the current level of importation of RSV infections.

For the reemergent epidemics in 2021 and 2022, our model (under Scenario 4) suggested an older mean age of hospitalizations, which was similar to the reported median patient age in Australia both before the pandemic and during the reemergent RSV epidemic.^47^ This makes intuitive sense, since many children born in 2020 were spared from RSV infection due to the low virus activity; these children will be older when they get infected for the first time during the reemergent epidemics. Consequently, stakeholders should consider modifying prophylaxis guidelines to include high-risk infants less than 2 years of age for the 2021 to 2022 season.

### Limitations

There are several limitations to our study. First, we did not have explicit data on the level of virus introduction or the effects of lack of boosting on the duration of protection provided to infants by transplacentally acquired antibodies to RSV. Although we explored a range of values, it is possible that the real values are outside of the range in our models. Our model estimations are most sensitive to the level of RSV introduction. Additional data on these factors will be helpful for future modeling of RSV transmission dynamics. Research on the relationship between virus exposure and the duration of transplacentally acquired immunity in infants may also help to explain discrepancies in the efficacy of maternal vaccines across different transmission settings.^48^ Second, we used historical inpatient data to fit our transmission models. The intensity and seasonality of RSV epidemics may have changed over the past few years. To address this possibility, we explored a variety of intensity levels and both annual and biennial cycles with data from different states in sensitivity analyses (see eFigure 9-18 in the Supplement). Also, ongoing RSV surveillance data from California,^49^ Florida,^37^ Minnesota,^50^ Oregon,^51^ and Texas^52^ suggests that RSV activity has been very consistent over recent years. Finally, there are other possible factors that could influence RSV epidemic dynamics over the coming years, particularly if any vaccines or long-lasting antibodies are recommended for general use.

## Conclusions

In this simulation modeling study, reemergent RSV epidemics in 2021 to 2022 were expected to be more intense and to affect patients in a broader age range than in typical RSV seasons. The timing of reemergent RSV epidemics might be different from the usual RSV season, depending on the duration of mitigation measures and the extent of virus introduction from other regions. Clinicians should be alert to the possibility of out-of-season RSV outbreaks.

## Notes

Supplement. **eAppendix.** Transmission Dynamic Models **eFigure 1.** Transmission Dynamic Model for RSV **eTable 1.** Shared Transmission Dynamic Model Parameters **eTable 2.** State-Specific Estimated Transmission Dynamic Model Parameters **eFigure 2.** Model Fit to Monthly RSV Hospitalization Data for New York **eFigure 3.** Model Fit to Monthly RSV Hospitalization Data for California **eFigure 4.** The Impact of Changes to the External Introduction of RSV Infections on RSV Epidemics **eFigure 5.** Simulated RSV Hospitalizations Using Parameters That Were Estimated From Historical Inpatient Data From Florida, 2019–2024 **eFigure 6.** Age Distribution of RSV Infections, Lower Respiratory Infections (LRIs) and Hospitalizations, 2021-2022 RSV Season **eFigure 7.** Expected RSV Hospitalizations Under Different Stringency of Mitigation Measures, New York, 2019–2025 **eFigure 8.** The Impact of Changes to the Duration of Transplacentally Acquired Immunity in Infants on RSV Epidemics **eFigure 9.** The Impact of Changes to the Length of Mitigation Measures on RSV Epidemics **eFigure 10.** Expected RSV Hospitalizations Under Different Scenarios, California, 2019–2025 **eFigure 11.** The Average Age of Hospitalization Among Children Under 5 Under Scenario 2, California **eFigure 12.** Age Distribution of RSV Infections, Lower Respiratory Infections (LRIs) and Hospitalizations, California, 2021-2022 RSV Season **eFigure 13.** Expected RSV hospitalizations under different scenarios for biennial epidemics peaking in even years, 2019–2025 **eFigure 14.** The Average Age of RSV Hospitalization for Biennial Epidemics Peaking in Even Years Among Children Under 5 **eFigure 15.** Age Distribution of RSV Infections, Lower Respiratory Infections (LRIs) and Hospitalizations Under the Assumption That Biennial Epidemics Are Greater in Even Years, 2021-2022 RSV Season **eFigure 16.** Expected RSV hospitalizations under different scenarios for biennial epidemics peaking in odd years, 2019–2025 **eFigure 17.** The Average Age of RSV Hospitalization for Biennial Epidemics Peaking in Odd Years Among Children Under 5 **eFigure 18.** Age Distribution of RSV Infections, Lower Respiratory Infections (LRIs) and Hospitalizations Under the Assumption That Biennial Epidemics Are Greater in Odd Years, 2021-2022 RSV Season
eReferences
