# Supplementary material for: Estimation of the Timing and Intensity of Reemergence of Respiratory Syncytial Virus Following the COVID-19 Pandemic in the US
Source: JAMA Netw Open. 2021 Dec 16;4(12):e2141779. doi: 10.1001/jamanetworkopen.2021.41779 (PMC8678706; doi:10.1001/jamanetworkopen.2021.41779)
Supplement: Supplement. — eAppendix. Transmission Dynamic Models eFigure 1. Transmission Dynamic Model for RSV eTable 1. Shared Transmission Dynamic Model Parameters eTable 2. State-Specific Estimated Transmission Dynamic Model Parameters eFigure 2. Model Fit to Monthly RSV Hospitalization Data for New York eFigure 3. Model Fit to Monthly RSV Hospitalization Data for California eFigure 4. The Impact of Changes to the External Introduction of RSV Infections on RSV Epidemics eFigure 5. Simulated RSV Hospitalizations Using Parameters That Were Estimated From Historical Inpatient Data From Florida, 2019–2024 eFigure 6. Age Distribution of RSV Infections, Lower Respiratory Infections (LRIs) and Hospitalizations, 2021-2022 RSV Season eFigure 7. Expected RSV Hospitalizations Under Different Stringency of Mitigation Measures, New York, 2019–2025 eFigure 8. The Impact of Changes to the Duration of Transplacentally Acquired Immunity in Infants on RSV Epidemics eFigure 9. The Impact of Changes to the Length of Mitigation Measures on RSV Epidemics eFigure 10. Expected RSV Hospitalizations Under Different Scenarios, California, 2019–2025 eFigure 11. The Average Age of Hospitalization Among Children Under 5 Under Scenario 2, California eFigure 12. Age Distribution of RSV Infections, Lower Respiratory Infections (LRIs) and Hospitalizations, California, 2021-2022 RSV Season eFigure 13. Expected RSV hospitalizations under different scenarios for biennial epidemics peaking in even years, 2019–2025 eFigure 14. The Average Age of RSV Hospitalization for Biennial Epidemics Peaking in Even Years Among Children Under 5 eFigure 15. Age Distribution of RSV Infections, Lower Respiratory Infections (LRIs) and Hospitalizations Under the Assumption That Biennial Epidemics Are Greater in Even Years, 2021-2022 RSV Season eFigure 16. Expected RSV hospitalizations under different scenarios for biennial epidemics peaking in odd years, 2019–2025 eFigure 17. The Average Age of RSV Hospitalization for Biennial Epidemics Peaking [file jamanetwopen-e2141779-s001.pdf]

Supplemental Online Content

Zheng Z, Pitzer VE, Shapiro ED, Bont LJ, Weinberger DM. Estimation of the timing and intensity of reemergence of respiratory syncytial virus following the COVID-19 pandemic in the US. *JAMA Netw Open.* 2021;4(12):e2141779. doi:10.1001/jamanetworkopen.2021.41779

eAppendix. Transmission Dynamic Models

eFigure 1. Transmission Dynamic Model for RSV

eTable 1. Shared Transmission Dynamic Model Parameters

eTable 2. State-Specific Estimated Transmission Dynamic Model Parameters

eFigure 2. Model Fit to Monthly RSV Hospitalization Data for New York

eFigure 3. Model Fit to Monthly RSV Hospitalization Data for California

eFigure 4. The Impact of Changes to the External Introduction of RSV Infections on RSV Epidemics

eFigure 5. Simulated RSV Hospitalizations Using Parameters That Were Estimated From Historical Inpatient Data From Florida, 2019–2024

eFigure 6. Age Distribution of RSV Infections, Lower Respiratory Infections (LRIs) and Hospitalizations, 2021-2022 RSV Season

eFigure 7. Expected RSV Hospitalizations Under Different Stringency of Mitigation Measures, New York, 2019–2025

eFigure 8. The Impact of Changes to the Duration of Transplacentally Acquired Immunity in Infants on RSV Epidemics

eFigure 9. The Impact of Changes to the Length of Mitigation Measures on RSV Epidemics

eFigure 10. Expected RSV Hospitalizations Under Different Scenarios, California, 2019–2025

eFigure 11. The Average Age of Hospitalization Among Children Under 5 Under Scenario 2, California

eFigure 12. Age Distribution of RSV Infections, Lower Respiratory Infections (LRIs) and Hospitalizations, California, 2021-2022 RSV Season

© 2021 Zheng Z et al. *JAMA Network Open.*

eFigure 13. Expected RSV hospitalizations under different scenarios for biennial epidemics peaking in even years, 2019–2025

eFigure 14. The Average Age of RSV Hospitalization for Biennial Epidemics Peaking in Even Years Among Children Under 5

eFigure 15. Age Distribution of RSV Infections, Lower Respiratory Infections (LRIs) and Hospitalizations Under the Assumption That Biennial Epidemics Are Greater in Even Years, 2021-2022 RSV Season

eFigure 16. Expected RSV hospitalizations under different scenarios for biennial epidemics peaking in odd years, 2019–2025

eFigure 17. The Average Age of RSV Hospitalization for Biennial Epidemics Peaking in Odd Years Among Children Under 5

eFigure 18. Age Distribution of RSV Infections, Lower Respiratory Infections (LRIs) and Hospitalizations Under the Assumption That Biennial Epidemics Are Greater in Odd Years, 2021-2022 RSV Season

eReferences

This supplemental material has been provided by the authors to give readers additional information about their work.

eAppendix. Transmission dynamic models

Mathematical models were used to reproduce the annual RSV epidemics before the COVID-19 pandemic based on the inpatient data of New York (2005-2014) and California (2003-2011). Parameters to produce biennial RSV epidemics and year-round RSV activity were taken from models fit to similar datasets from Colorado (1989–2009) and Florida (1989–2009), respectively. This model assumes infants are born with transplacentally-acquired antibodies against RSV infections from their mothers (M). As transplacentally-acquired protective antibodies wane, infants become susceptible to infection (S_0). Following each infection (I_i), individuals gain partial immunity that lowers both their susceptibility to subsequent infections and the duration and infectiousness of subsequent infections (see eFigure 1). The force of infection for a specific age group a, $\lambda_a(t)$, for time *t* is defined as:

$$\lambda_a(t) = \left(1 + b_1 \cos\left(\frac{2\pi t - \phi}{12}\right)\right) \sum_k \beta_{a,k}(I_{1,k}(t) + \rho_1 I_{2,k}(t) + \rho_2 I_{3,k}(t) + \rho_2 I_{4,k}(t))/N_k(t)$$

Seasonality in the force of infection is represented by $(1 + b_1 \cos(\frac{2\pi t - \phi}{12}))$, where b_1 is the amplitude of seasonality and ϕ is the seasonal offset. The chance of susceptible individuals in age group a being infected is influenced by their contacts with infectious individuals in the entire population. $\beta_{a,k}$ is the transmission rate from age group *k* to age group a. The proportion of infected individuals and their relative infectiousness at time *t* is denoted by $(I_{1,k}(t) + \rho_1 I_{2,k}(t) + \rho_2 I_{3,k}(t) + \rho_2 I_{4,k}(t))/N_k(t)$, where $I_{1,k}$ is the number of infectious individuals of age *k* during their first infection; $I_{2,k}$, $I_{3,k}$ and $I_{4,k}$ are the number of infectious individuals who have been infected two, three and four or more times, respectively; ρ_1 and ρ_2 denote the relative infectiousness of the second and subsequent infections; and N_k is the total population of age *k*.

The transmission parameter $\beta_{a,k}$ can be further decomposed into the age-specific contact probability between age group a and k per unit time ($C_{a,k}$) and the probability of transmission given contact between an infectious and a susceptible individual (q). Age-specific mixing patterns were obtained from several previous studies, including detailed contact patterns for infants under 1 year of age and location-specific contact patterns.[1-3] Age was stratified into thirteen groups: infants younger than 3 months, 3-5 months, 6-8 months, 9-11 months, 1 year, 2 years, 3 years, 4 years, 5-9 years, 10-19 years, 20-39 years, 40-59 years, and ≥60 years.

The disease transmission process is linked to observation-level information. The probabilities of developing lower respiratory tract disease and being hospitalized upon RSV infection are informed by cohort studies conducted in the US and Kenya.[1,4-14] The number of lower respiratory tract infections (LRI) due to RSV is given by:

$$D_a(t) = \lambda_a(t)(S_{0,a}(t)d_{1,a} + \sigma_1 S_{1,a}(t)d_{2,a} + \sigma_2 S_{2,a}(t)d_{3,a} + \sigma_3 S_{3,a}(t)d_{3,a})$$

while the number of hospitalizations is given by:

$$H_a(t) = \lambda_a(t)(S_{0,a}(t)h_{1,a} + \sigma_1 S_{1,a}(t)h_{2,a} + \sigma_2 S_{2,a}(t)h_{3,a} + \sigma_3 S_{3,a}(t)h_{3,a})$$

where $\lambda_a(t)$ is the force of infection for a specific age group a at time *t* (as defined above). $S_{0,a}$ is the number of fully susceptible individuals of age *a*; $S_{1,a}$, $S_{2,a}$ and $S_{3,a}$ are the number of susceptible individuals who have been infected once, twice and more times, respectively. σ_1, σ_2 and σ_3 denote the relative risk of infection following the first, second, and more infections. $h_{1,a}$, $h_{2,a}$ and $h_{3,a}$ are the proportion of the first, second, and more infections that are hospitalized.

The average age of hospitalization among children under 5 in month *t* is given by:[15]

$$A(t) = \frac{\sum P_a \lambda_a(t)(S_{0,a}(t)h_{1,a} + \sigma_1 S_{1,a}(t)h_{2,a} + \sigma_2 S_{2,a}(t)h_{3,a} + \sigma_3 S_{3,a}(t)h_{3,a})}{\sum \lambda_a(t)(S_{0,a}(t)h_{1,a} + \sigma_1 S_{1,a}(t)h_{2,a} + \sigma_2 S_{2,a}(t)h_{3,a} + \sigma_3 S_{3,a}(t)h_{3,a})}$$

where the weight P_a is the midpoint of age group a.

© 2021 Zheng Z et al. *JAMA Network Open.*

Several model parameters were fixed based on data from previous cohort and modeling studies.[1,4-14] We used Bayesian inference to estimate the average duration of transplacentally-acquired immunity, age-specific probability of hospitalization in the 40-59 year and >60 year age groups, the transmissibility coefficient, and seasonal parameters by fitting the model to the hospitalization data from New York and California.[16,17] We identified the best-fit parameter sets by maximum a posteriori estimation.[18] The likelihood was calculated by assuming the observed number of hospitalizations in the entire population was Poisson-distributed with a mean equal to the model-predicted number of hospitalization, and that the observed age distribution was multinomial-distributed with probabilities equal to the model-predicted distribution of RSV hospitalizations in each age group.

To validate our model predictions, we fitted the transmission model to the inpatient data for California from 2003 to 2011; we then compared the model predictions with data on the percent of clinical specimens positive for RSV from a separate sentinel surveillance database from 2012 to 2018. We rescaled the percent positive data by calculating a scaling factor based on overlaying the surveillance data and inpatient data from 2009 to 2011 (see eFigure 3).

We initialized the transmission models with 1 infectious individual in each age group (except for infants under 6 months) in July 1981 and used a burn-in period of 24 years and 22 years in New York and California, respectively. We also performed a sensitivity analysis around what re-emergence might look like in a state with a biennial pattern of epidemics, using parameters fitted to earlier data from Colorado as an example and assuming a linearly declining birth rate (from 17 to 10 births per 1,000 people per year). We used the same number of infectious individuals to initialize transmission model, and a burn-in period of 40 or 41 years starting from 1971 or 1970 to allow for greater incidence in even or odd years.

© 2021 Zheng Z et al. *JAMA Network Open.*

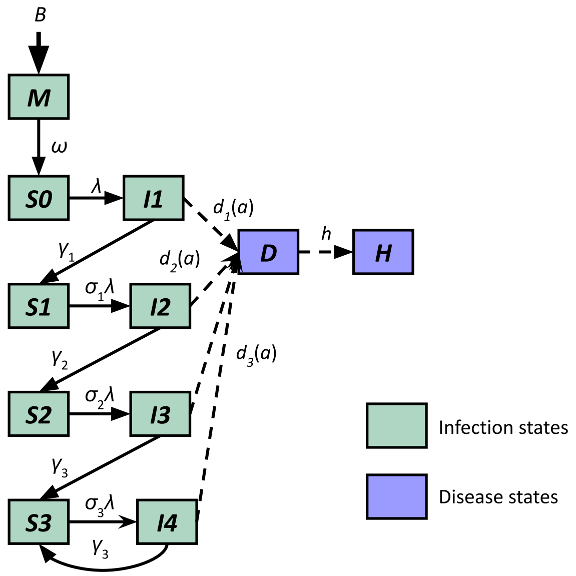

eFigure 1. Transmission dynamic model for RSV. The green boxes represent infection states in the model, while purple boxes represent diseased states (RSV lower respiratory illness, D, and RSV hospitalizations, H).

© 2021 Zheng Z et al. *JAMA Network Open.*

eTable 1. Shared transmission dynamic model parameters.

Parameter description	Symbol	Parameter value	Reference for fixed or prior value	Note
Duration of transplacentally acquired antibodies against RSV infections in infants	$1/\Omega$	112 days	[19]	Fitted for NY and CA using maximum a posteriori estimation, assuming a Gamma(10,11) prior distribution
Duration of infectiousness				
First infection	$1/\gamma_1$	10 days	[20]	
Second infection	$1/\gamma_2$	7 days		
Subsequent infection	$1/\gamma_3$	5 days		
Relative risk of infection following				
First infection	σ_1	0.76	[4,8,9,21]	
Second infection	σ_2	0.6		
Subsequent infection	σ_3	0.4		
Relative infectiousness				
Second infections	ρ_1	0.75	[4,8,10]	
Subsequent infections	ρ_2	0.51	[22]	
Proportion of RSV infections leading to lower respiratory tract infection				
First infection			[5] $\Pr(LRI\|I_s)$<br><br>[23] $\Pr(I_s\|I)$	The probability of lower respiratory infection (LRI) given infection was estimated as the product of LRI given symptomatic infection (I_S) times the probability of symptoms given infection: $\Pr(LRI\|I) = \Pr(LRI\|I_s) * \Pr(I_s\|I)$
0-2 months old	$d_{p,0-2}$	0.44*0.9		
3-5 months old	$d_{p,3-5}$	0.43*0.9		
6-8 months old	$d_{p,6-8}$	0.23*0.9		
9-11 months old	$d_{p,9-11}$	0.22*0.9		
1-2 years old	$d_{p,1}$	0.21*0.8		
2-4 years old	$d_{p,2}$	0.2*0.8		
≥ 5 years old	$d_{p,adults}$	0.05	[24]	
Second infection	$d_{s,a}$	0.5*$d_{p,a}$	[8]	
Third+ infection	$d_{t,a}$	0.7*$d_{s,a}$	[8]	
Proportion of RSV infections leading to hospitalization				
First infection, <3 months old	$h_{p,0-2}$	0.20*$d_{p,0-2}$	[27,28]	
3-5 months old	$h_{p,3-5}$	0.08*$d_{p,3-5}$	[10,25,26]	
6-8 months old	$h_{p,6-8}$	0.07*$d_{p,6-8}$		
9-11 months old	$h_{p,9-11}$	0.06*$d_{p,9-11}$		
1-2 years old	$h_{p,1}$	0.06*$d_{p,1}$		
2-4 years old	$h_{p,2}$	0.05*$d_{p,2-4}$		
≥ 5 years old	$h_{p,adults}$	0.02*$d_{p,adults}$		
Second infection	$h_{s,a}$	0.4*$H_{p,a}$	[8]	
Third infection	$h_{t,a}$	0 except for the elderly		Fitted for the elderly using maximum a posteriori estimation,

© 2021 Zheng Z et al. *JAMA Network Open.*

				assuming priors follow a uniform distribution U(0,1)
Scenarios for the impact of mitigation measures				
Reduction in RSV transmission		10%-25%	[29]	
Decrease in non-household contacts		82%	[30]	
Increase in household contacts		10%	[31]	
External seeding during control period		0%-66%	[32,33]	

© 2021 Zheng Z et al. *JAMA Network Open.*

eTable 2. State-specific estimated transmission dynamic model parameters.

	New York	California	Colorado	Florida
**Duration of maternal immunity**	116.05	76	112	112
**Basic reproductive number***	9.00	8.88	8.91	9.11
**Amplitude of seasonality**	0.16	0.25	0.24	0.11
**Timing of seasonality**	0.54	0.44	0.49	0.34[+]
**Reporting fraction**	1	0.40	1	0.6

*The basic reproductive number (R_0) was estimated from $R_0 = \frac{\det(\beta_{a,k})}{\gamma_1} = \frac{\det(qC_{a,k})}{\gamma_1}$, using the next-generation matrix method; the parameter *q* was fitted to the data.

[+]Timing of seasonality was adjusted from historical estimates for Florida because of the assumption of virus introduction.

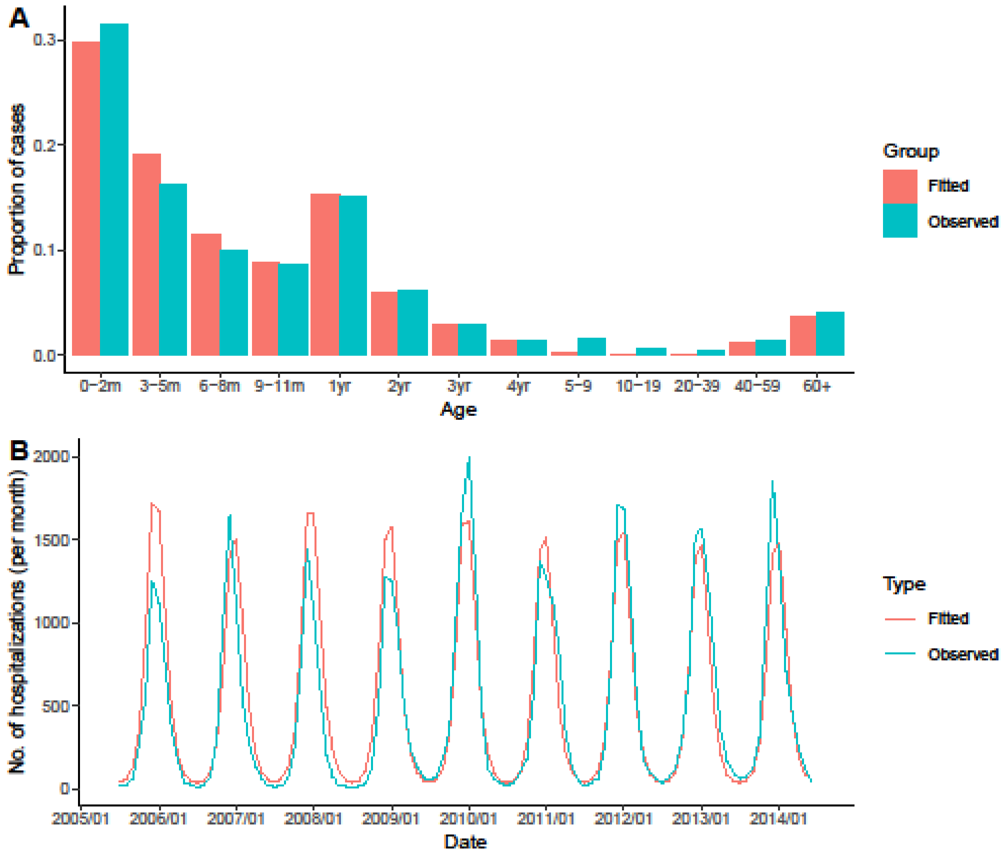

eFigure 2. Model fit to monthly RSV hospitalization data for New York. The ICD9-CM coded hospitalization data is shown in blue and the fitted models are shown in red.

© 2021 Zheng Z et al. *JAMA Network Open.*

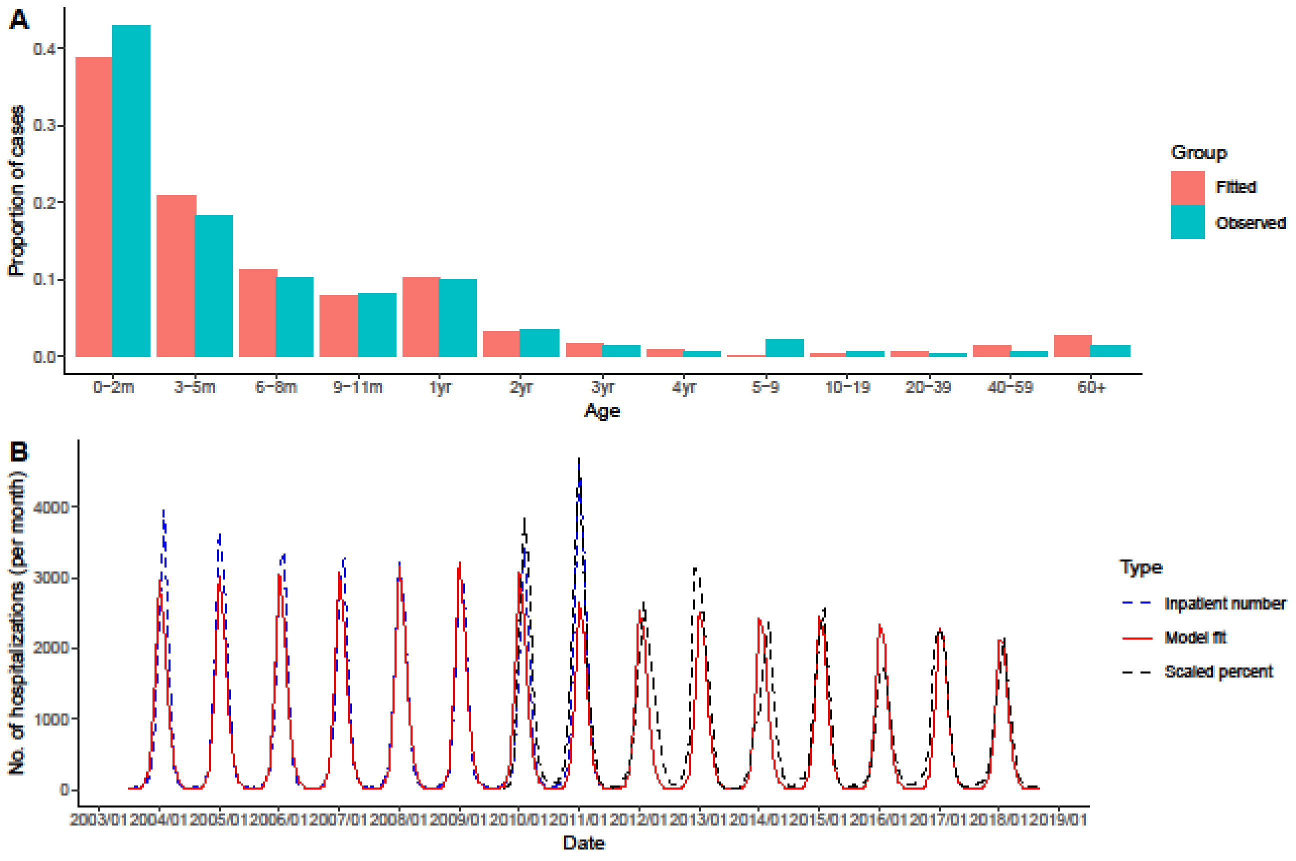

eFigure 3. Model fit to monthly RSV hospitalization data for California. The ICD9-CM coded hospitalization data is shown in blue; the rescaled RSV percent positive data is shown in black, and the fitted models are shown in red.

© 2021 Zheng Z et al. *JAMA Network Open.*

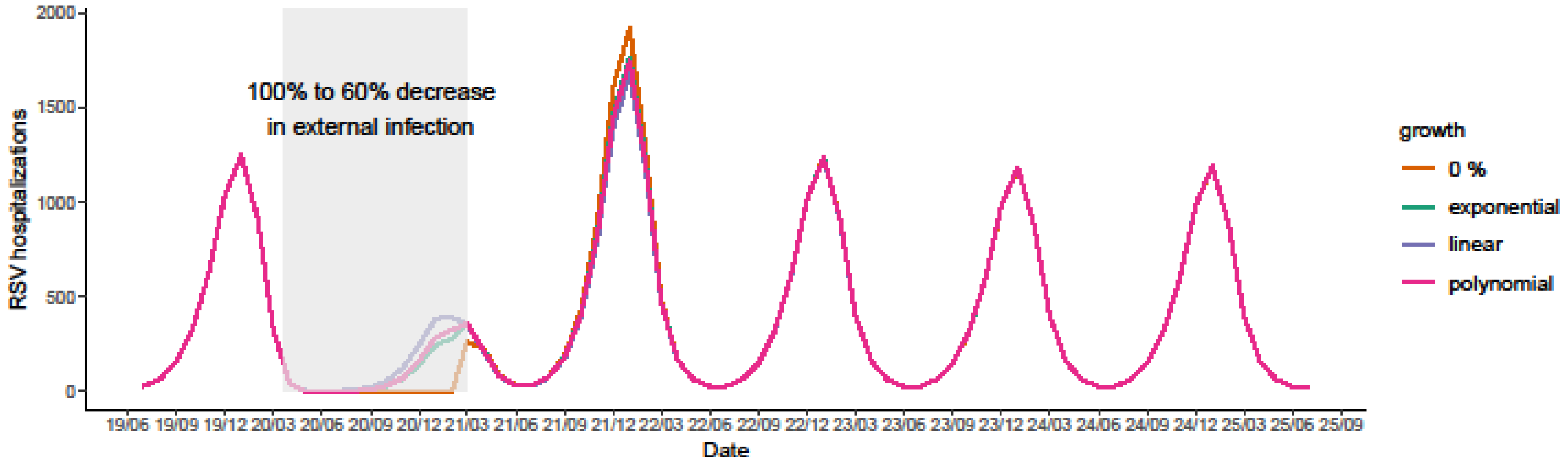

eFigure 4. The impact of changes to the external introduction of RSV infections on RSV epidemics. This plot assumed a background rate of external infections of 5/100,000 people per month. The grey area indicates decreased external infections because of mitigation measures. The orange line shows the RSV epidemics if there were not external infections during April 1, 2020 to March 1, 2021. The green line, purple line and pink lines indicate a sudden decrease in external infections in April 2020 followed by a gradual increase with different growth rates.

© 2021 Zheng Z et al. *JAMA Network Open.*

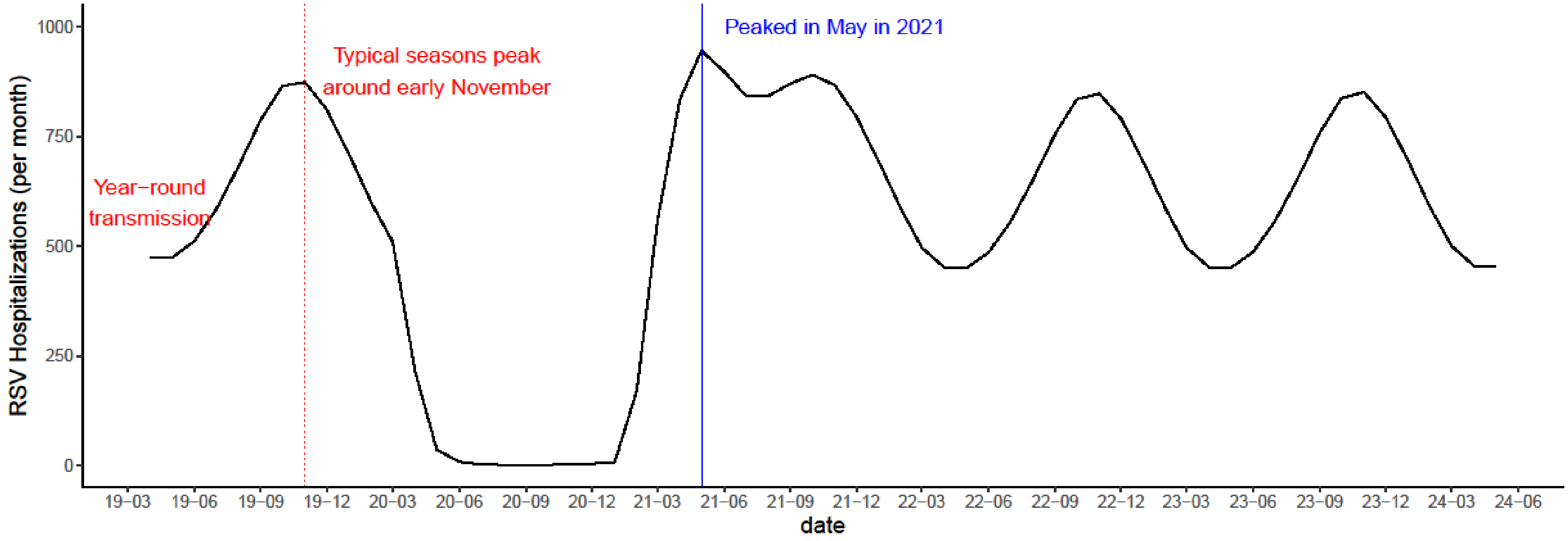

eFigure 5. Simulated RSV hospitalizations using parameters that were estimated from historical inpatient data from Florida, 2019–2024. The black time series shows the simulated RSV hospitalizations under the assumption of considerable virus introduction from other regions (30 infections/100,000 people per month). The dotted red vertical line shows the peak timing of RSV epidemics in a typical RSV season. The solid blue vertical line shows the peak timing of RSV epidemics in 2021. Compared with other U.S. states, Florida exhibits year-round transmission and weaker seasonality.

© 2021 Zheng Z et al. *JAMA Network Open.*

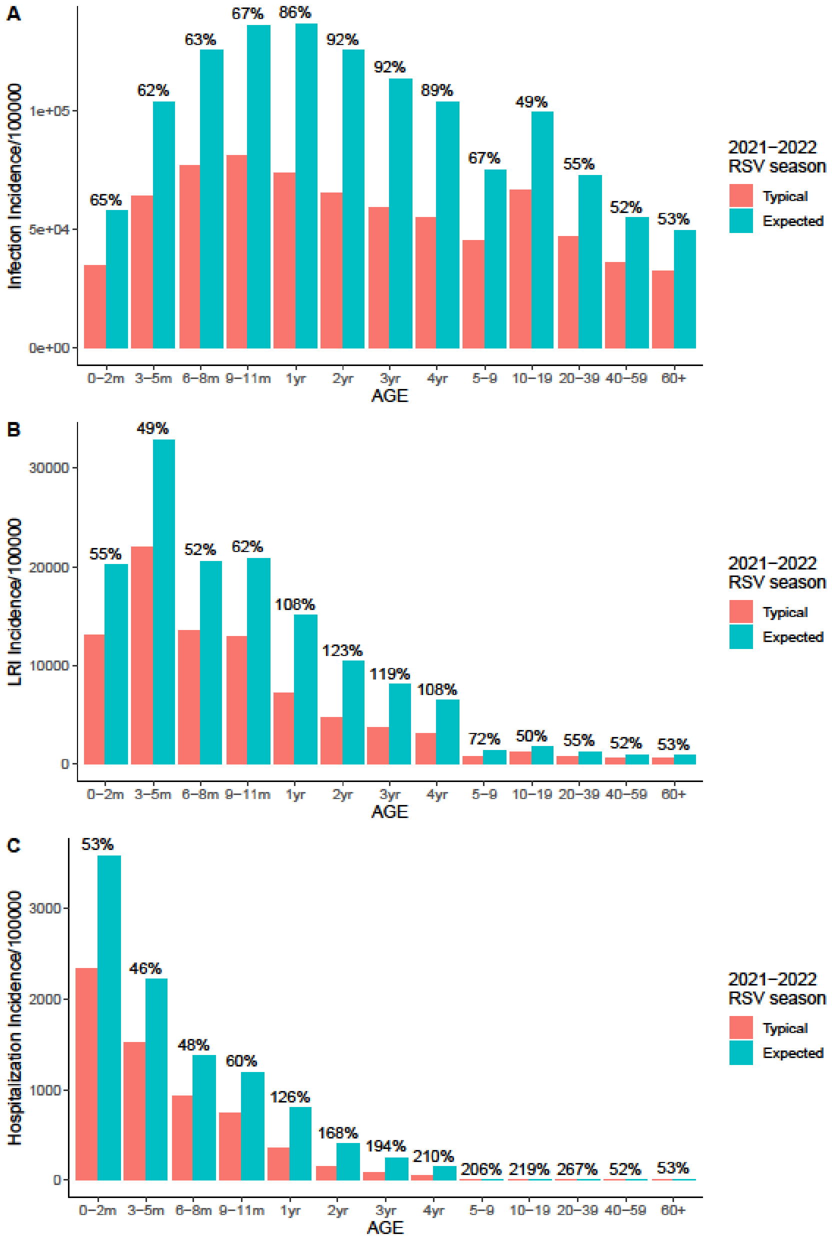

eFigure 6. Age distribution of RSV infections, lower respiratory infections (LRIs) and hospitalizations, 2021-2022 RSV season. Panels A to C correspond to RSV infections, RSV LRIs and RSV hospitalizations, respectively. The red bars show the counterfactual incidence of RSV cases during the 2021-2022 RSV season if there was no COVID-19 pandemic and no mitigation 574 measures in place. The blue bars show the expected incidence of RSV cases under Scenario 2 during the 2021-2022 RSV season. The numbers on the top show the percentage difference between the expected incidence and the counterfactual incidence in each age group.

© 2021 Zheng Z et al. *JAMA Network Open.*

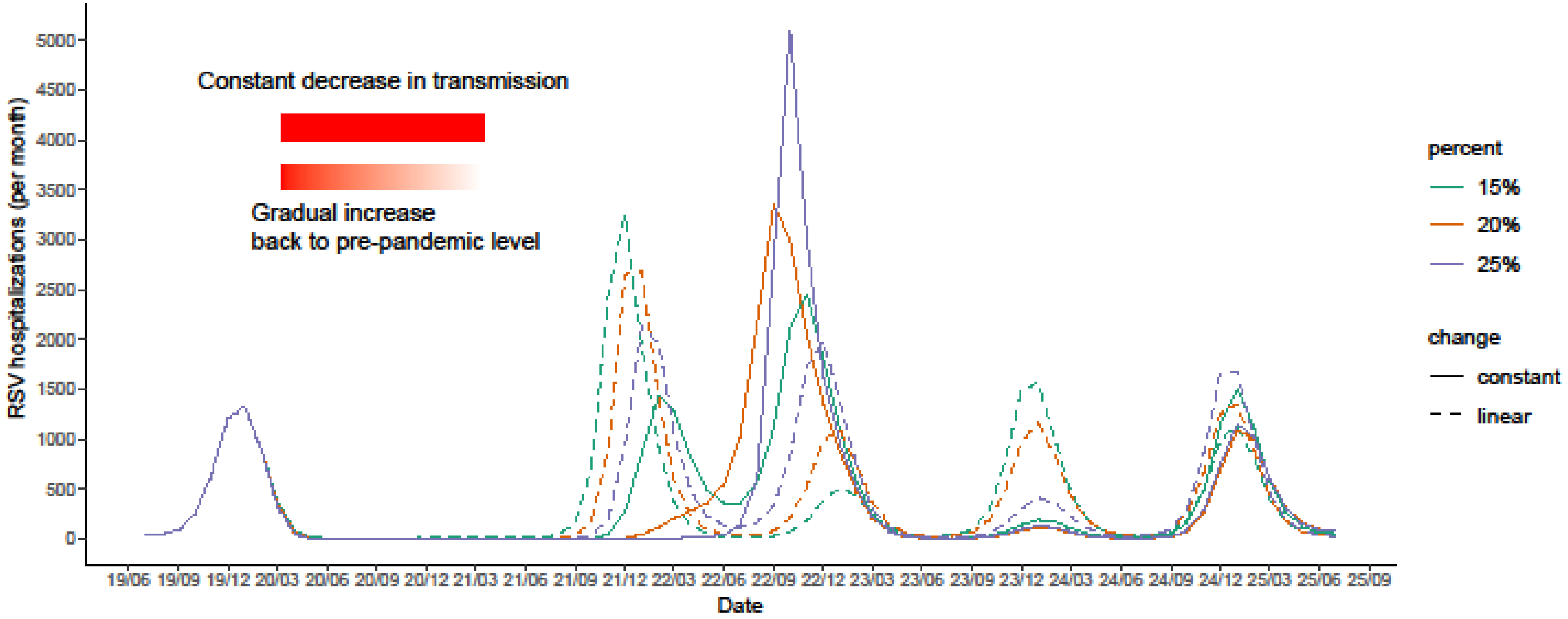

eFigure 7. Expected RSV hospitalizations under different stringency of mitigation measures, New York, 2019–2025. The color lines indicate the percentage decrease under either constant decrease or linear change scenario. The solid lines represent a constant decrease in transmission. The dash lines represent the RSV hospitalization incidence under the assumption that mitigation measures are most strict at the beginning and are gradually relaxed. The solid red rectangle and the gradient red rectangle indicate the length of the change period is from March 2020 to March 2021.

© 2021 Zheng Z et al. *JAMA Network Open.*

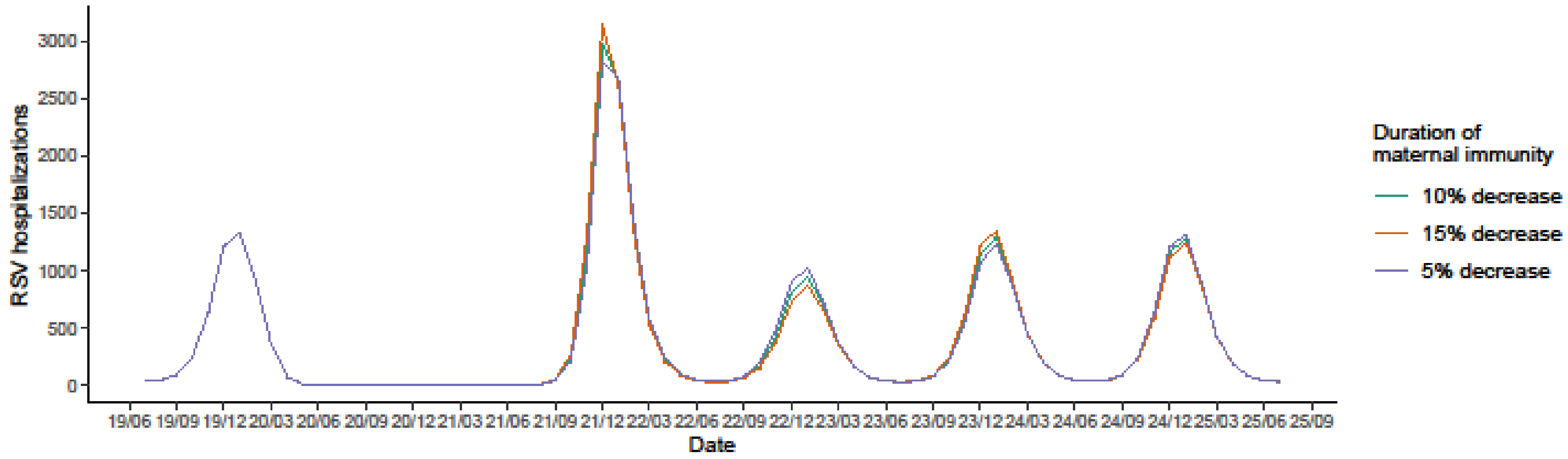

eFigure 8. The impact of changes to the duration of transplacentally acquired immunity in infants on RSV epidemics. The colors of the lines show different percentage decrease in the duration of transplacental -acquired immunity in infants as a result of lack of boosting in pregnant women.

© 2021 Zheng Z et al. *JAMA Network Open.*

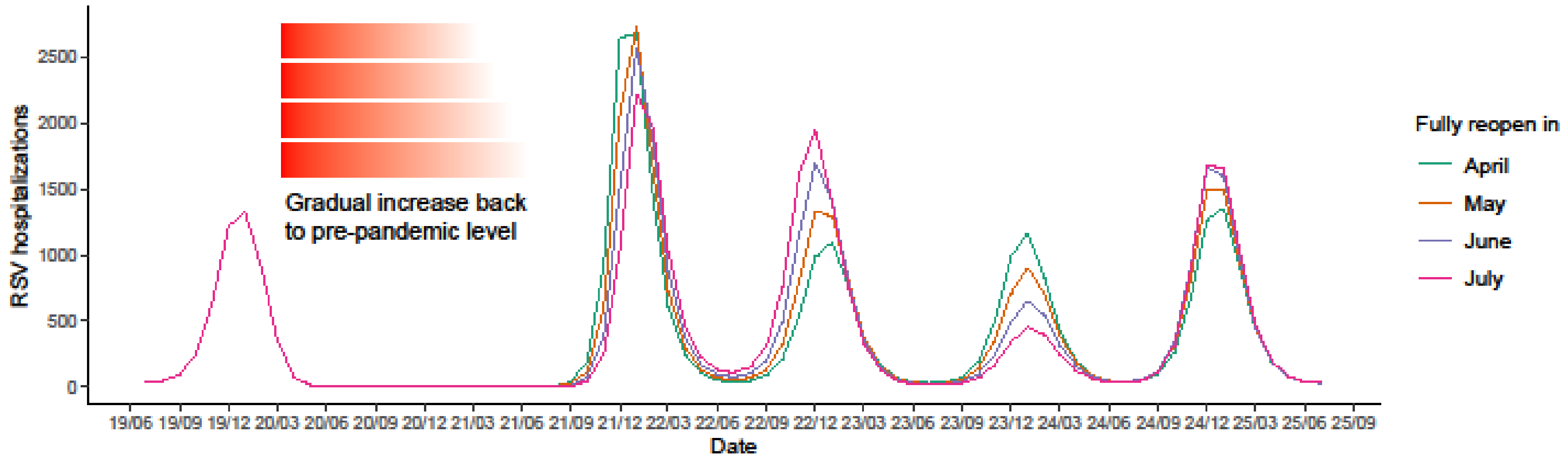

eFigure 9. The impact of changes to the length of mitigation measures on RSV epidemics. The colors of the lines show the expected RSV epidemics with different reopening dates in 2021 (first day of the month). For these scenarios, we assume a sudden decrease in transmission in March 2020 followed by a linear increase back to pre-pandemic levels.

© 2021 Zheng Z et al. *JAMA Network Open.*

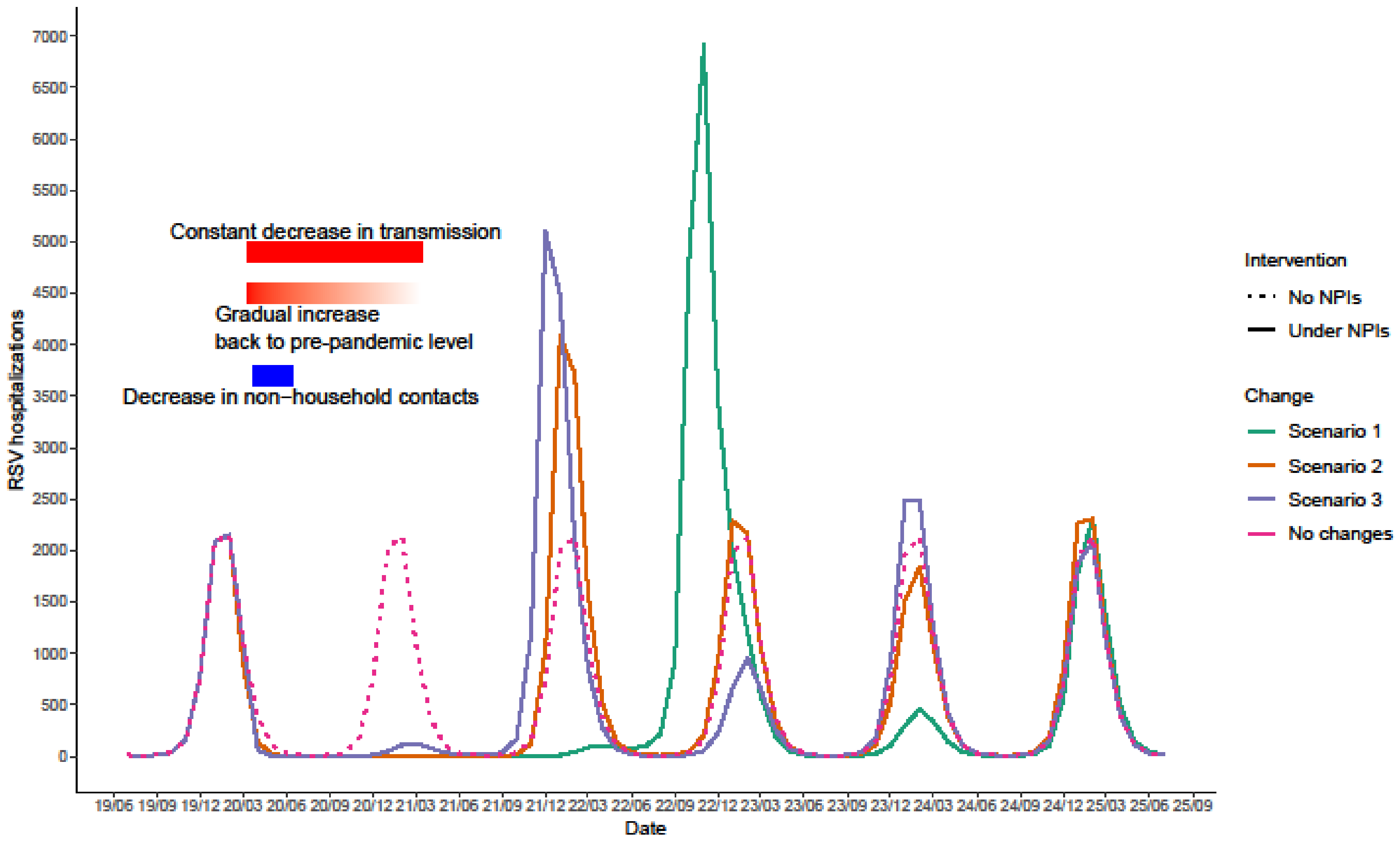

eFigure 10. Expected RSV hospitalizations under different scenarios, California, 2019–2025. The dotted dark pink line shows the counterfactual scenario that there is no COVID-19 pandemic and no mitigation measures in place. The solid lines show three scenarios of stringency of mitigation measures. The green line represents Scenario 1: 20% constant decreased transmission from March 2020 to March 2021. The orange line represents Scenario 2: a sudden 20% decrease in RSV transmission in March 2020 followed by a linear increase back to normal. The purple line represents Scenario 3: 82% decreased non-household contacts and 10% increased household contacts between April and July 2020. The red rectangle on the top, the gradient red rectangle in the middle and the blue rectangle on the bottom indicate the length and the stringency of Scenario 1, Scenario 2 and Scenario 3, respectively.

© 2021 Zheng Z et al. *JAMA Network Open.*

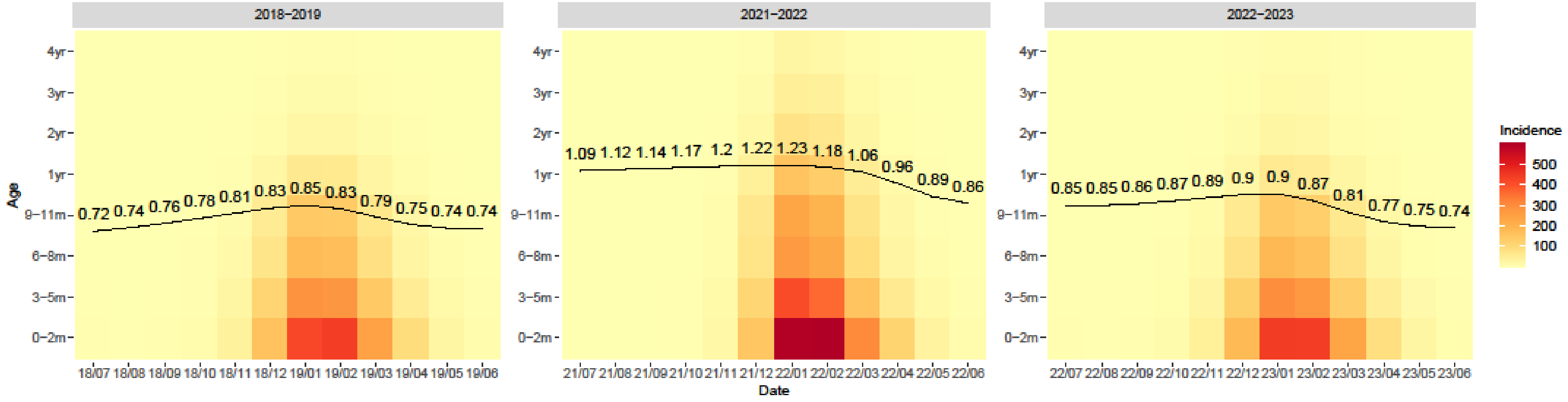

eFigure 11. The average age of hospitalization among children under 5 under Scenario 2, California. The background color represents the incidence of RSV hospitalization per 100,000 people per month in each age group in each month. The darker color suggests a higher incidence. The black line and value indicate the average age of hospitalization (in years) varies with time.

© 2021 Zheng Z et al. *JAMA Network Open.*

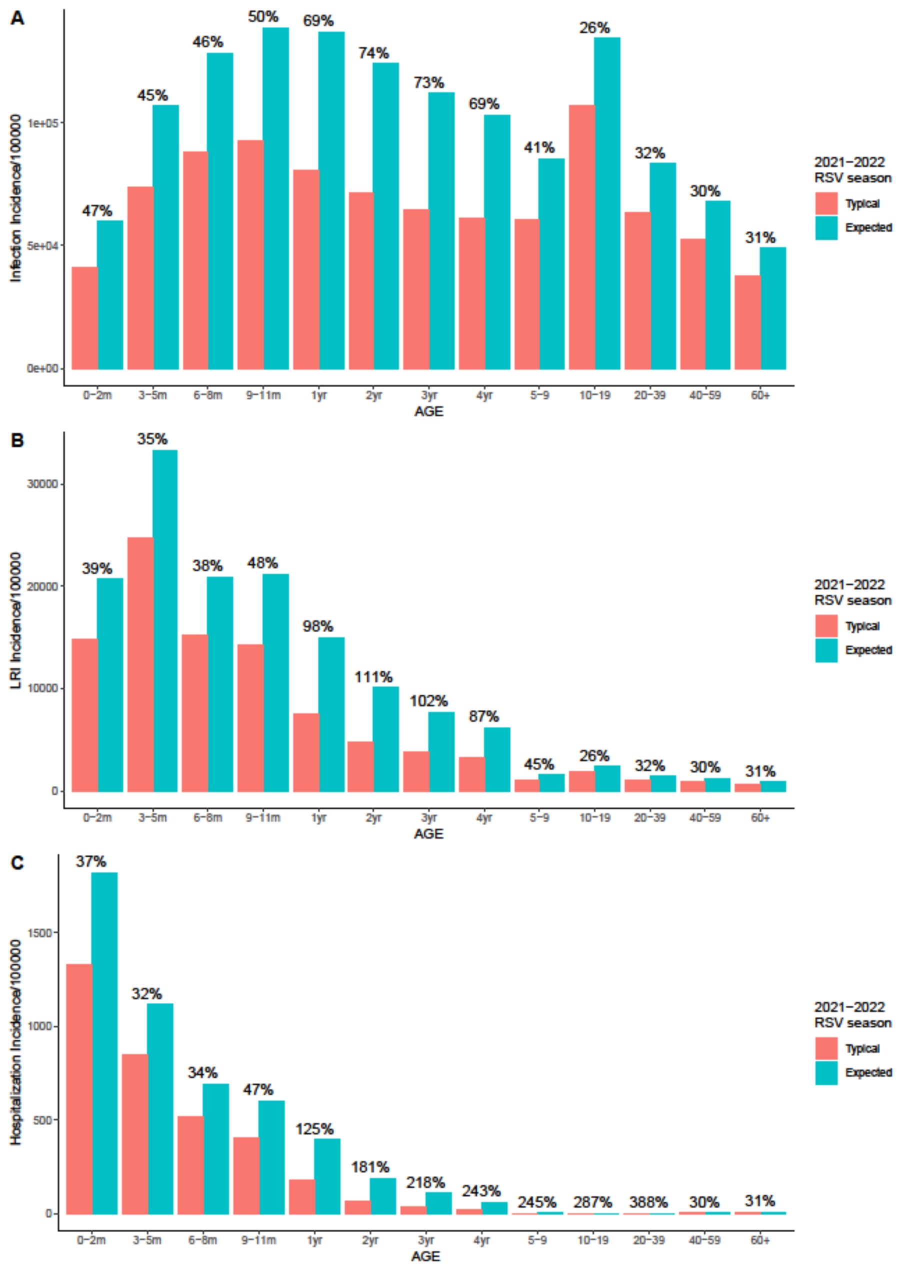

eFigure 12. Age distribution of RSV infections, lower respiratory infections (LRIs) and hospitalizations, California, 2021-2022 RSV season. Panel A to C correspond to RSV infections, RSV LRIs and RSV hospitalizations. The red color bars show the counterfactual incidence of RSV cases during the 2021-2022 RSV season if there was no COVID-19 pandemic and no mitigation measures in place. The blue color bars show the expected incidence of RSV cases under Scenario 2 during the 2021-2022 RSV season. The numbers on the top show the percentage difference between the expected incidence and the counterfactual incidence in each age group.

© 2021 Zheng Z et al. *JAMA Network Open.*

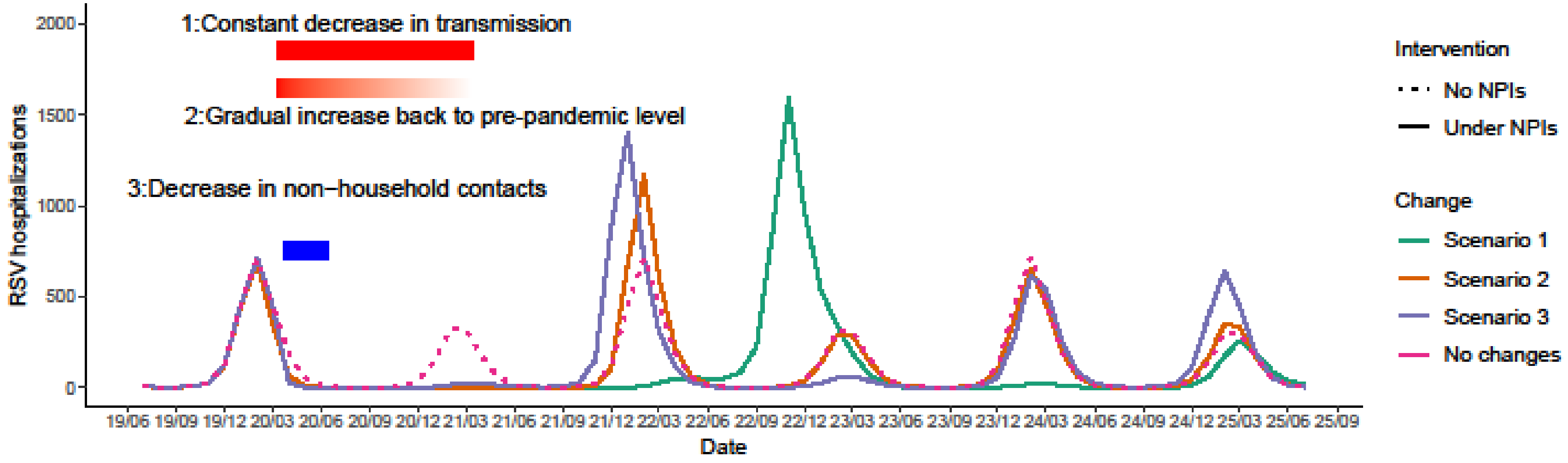

eFigure 13. Expected RSV hospitalizations under different scenarios for biennial epidemics peaking in even years, 2019–2025. The dotted dark pink line shows the counterfactual scenario that there is no COVID-19 pandemic and no mitigation measures in place. The solid lines show three scenarios of stringency of mitigation measures. The green line represents Scenario 1: 20% constant decreased transmission from March 2020 to March 2021. The orange line represents Scenario 2: a sudden 20% decrease in RSV transmission in March 2020 followed by a linear increase back to normal. The purple line represents Scenario 3: 82% decreased non-household contacts and 10% increased household contacts between April and July 2020. The red rectangle on the top, the gradient red rectangle in the middle and the blue rectangle on the bottom indicate the length and the stringency of Scenario 1, Scenario 2 and Scenario 3, respectively.

© 2021 Zheng Z et al. *JAMA Network Open.*

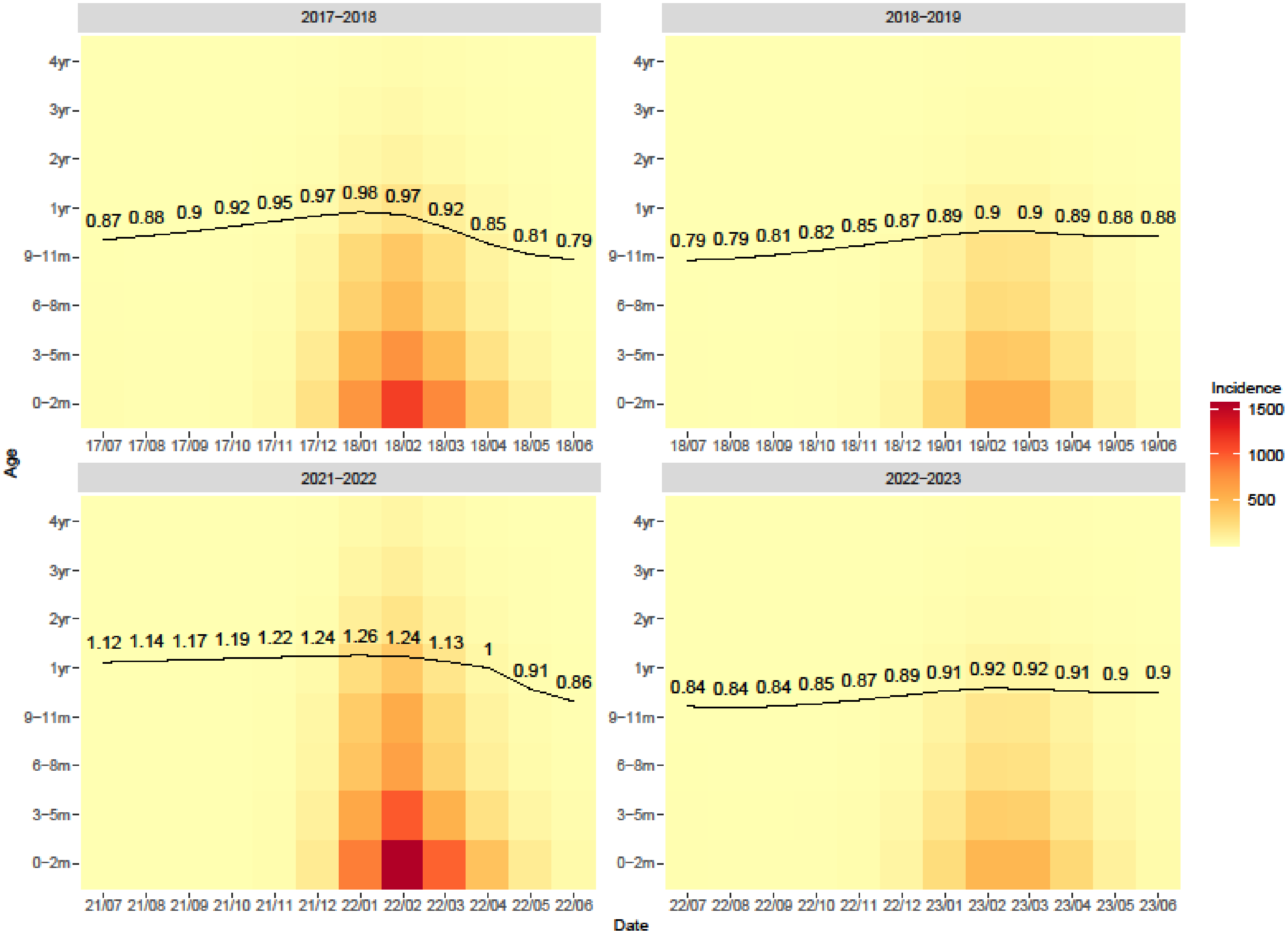

eFigure 14. The average age of RSV hospitalization for biennial epidemics peaking in even years among children under 5. The background color represents the incidence of RSV hospitalization per 100000 per month in each age group in each month. The darker color suggests a higher incidence. The black line and value indicate the average age of hospitalization (in years) varies with time.

© 2021 Zheng Z et al. *JAMA Network Open.*

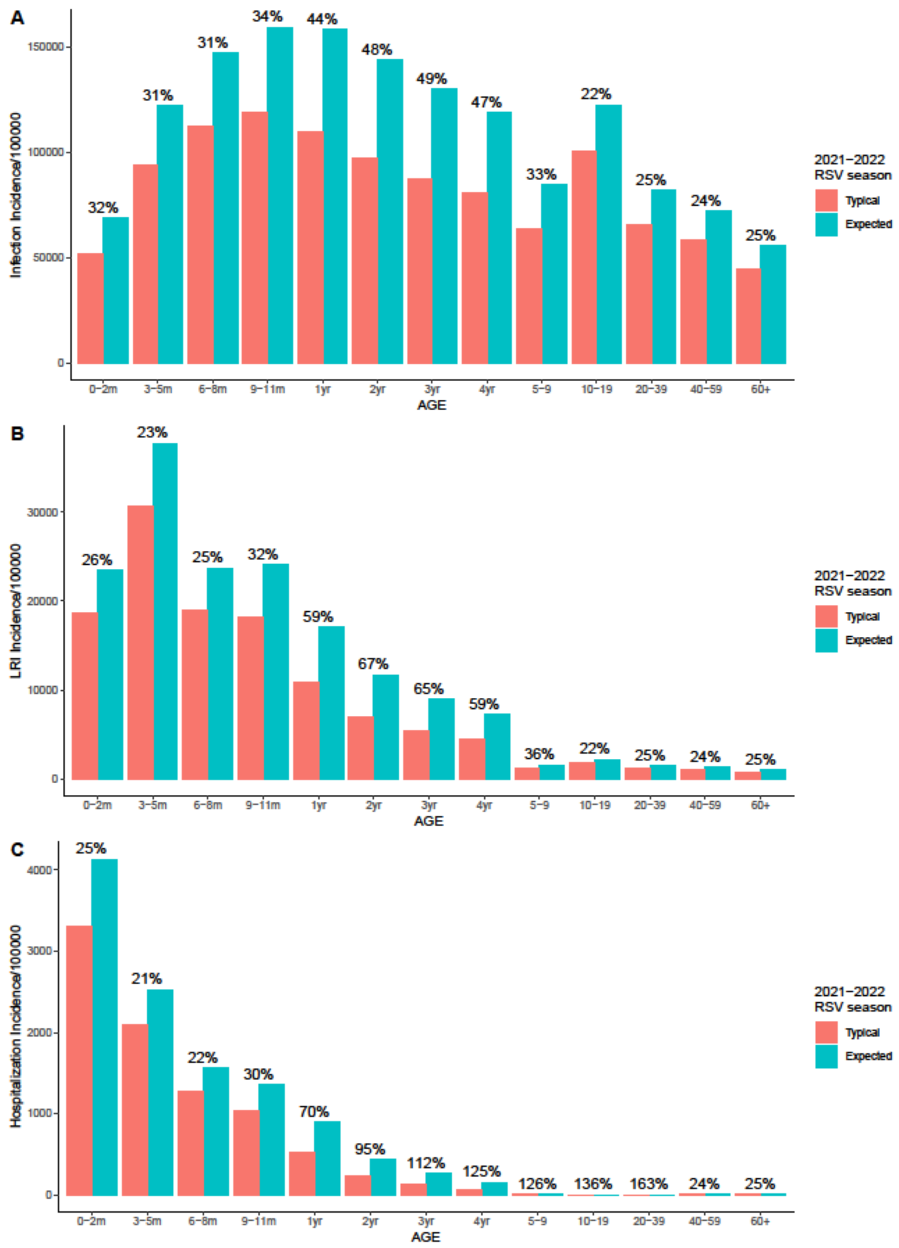

eFigure 15. Age distribution of RSV infections, lower respiratory infections (LRIs) and hospitalizations under the assumption that biennial epidemics are greater in even years, 2021-2022 RSV season. Panel A to C correspond to RSV infections, RSV LRIs and RSV hospitalizations. The red color bars show the counterfactual incidence of RSV cases during 2021 to 2022 RSV season if there was no COVID-19 pandemic and no mitigation measures in place. The blue color bars show the expected incidence of RSV cases under Scenario 2 during the 2021-2022 RSV season. The numbers on the top show the percentage difference between the expected incidence and the counterfactual incidence in each age group.

© 2021 Zheng Z et al. *JAMA Network Open.*

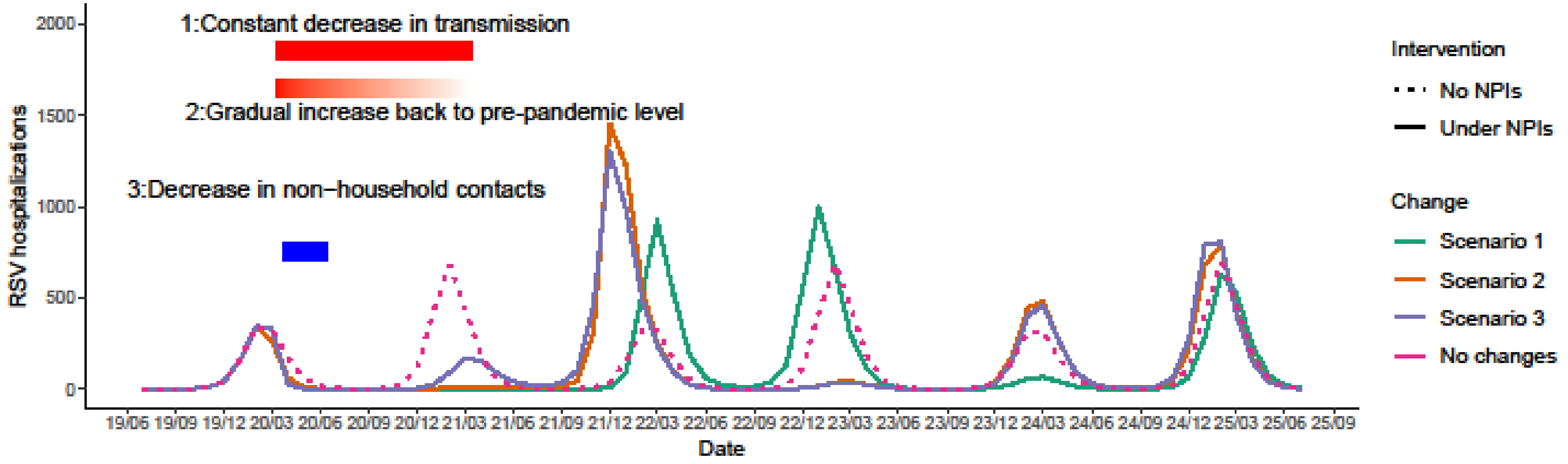

eFigure 16. Expected RSV hospitalizations under different scenarios for biennial epidemics peaking in odd years, 2019–2025. The dotted dark pink line shows the counterfactual scenario that there is no COVID-19 pandemic and no mitigation measures in place. The solid lines show three scenarios of stringency of mitigation measures. The green line represents Scenario 1: 20% constant decreased transmission from March 2020 to March 2021. The orange line represents Scenario 2: a sudden 20% decrease in RSV transmission in March 2020 followed by a linear increase back to normal. The purple line represents Scenario 3: 82% decreased non-household contacts and 10% increased household contacts between April and July 2020. The red rectangle on the top, the gradient red rectangle in the middle and the blue rectangle on the bottom indicate the length and the stringency of Scenario 1, Scenario 2 and Scenario 3, respectively.

© 2021 Zheng Z et al. *JAMA Network Open.*

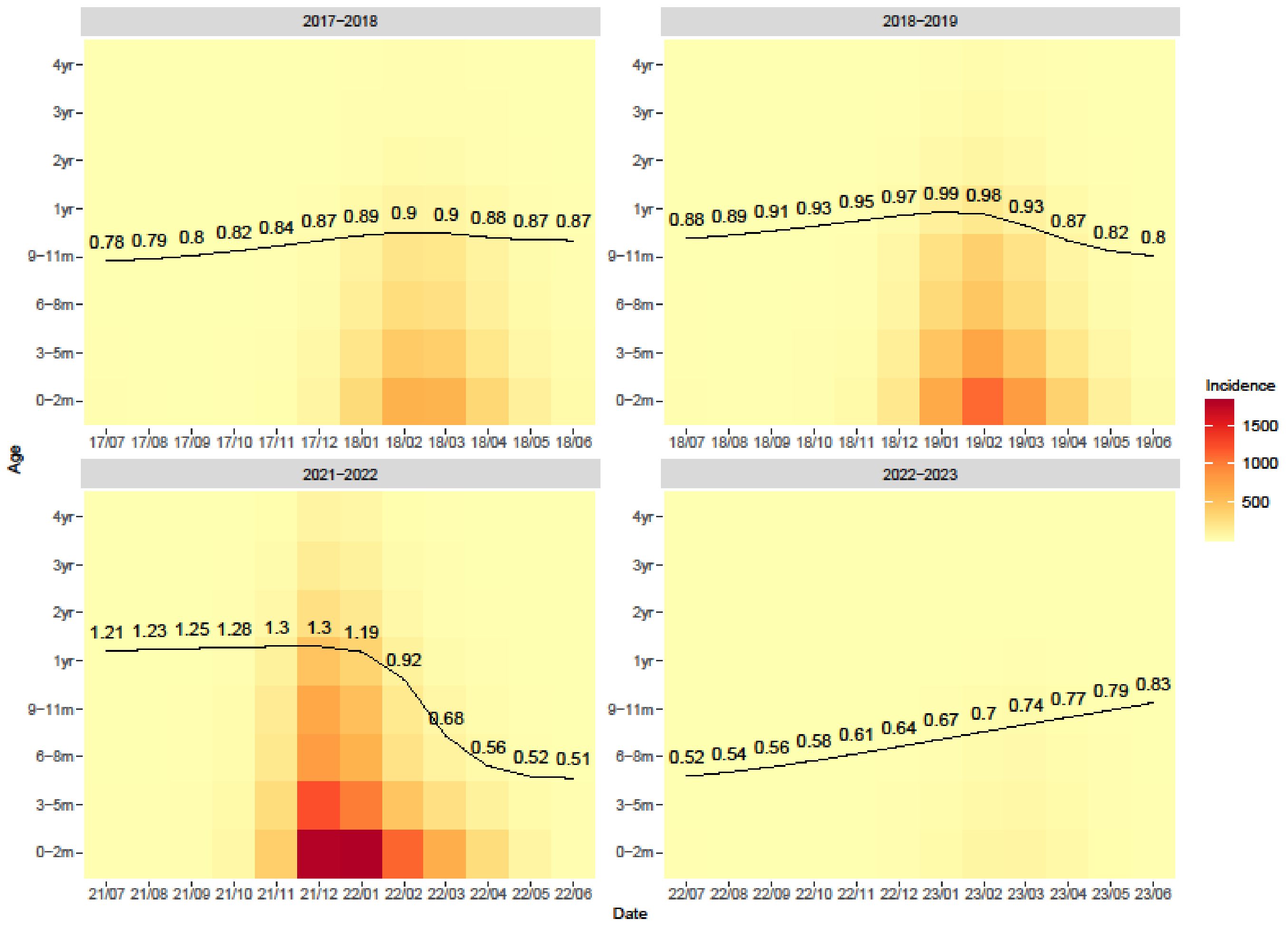

eFigure 17. The average age of RSV hospitalization for biennial epidemics peaking in odd years among children under 5. The background color represents the incidence of RSV hospitalization per 100000 people per month in each age group in each month. The darker color suggests a higher incidence. The black line and value indicate the average age of hospitalization (in years) varies with time.

© 2021 Zheng Z et al. *JAMA Network Open.*

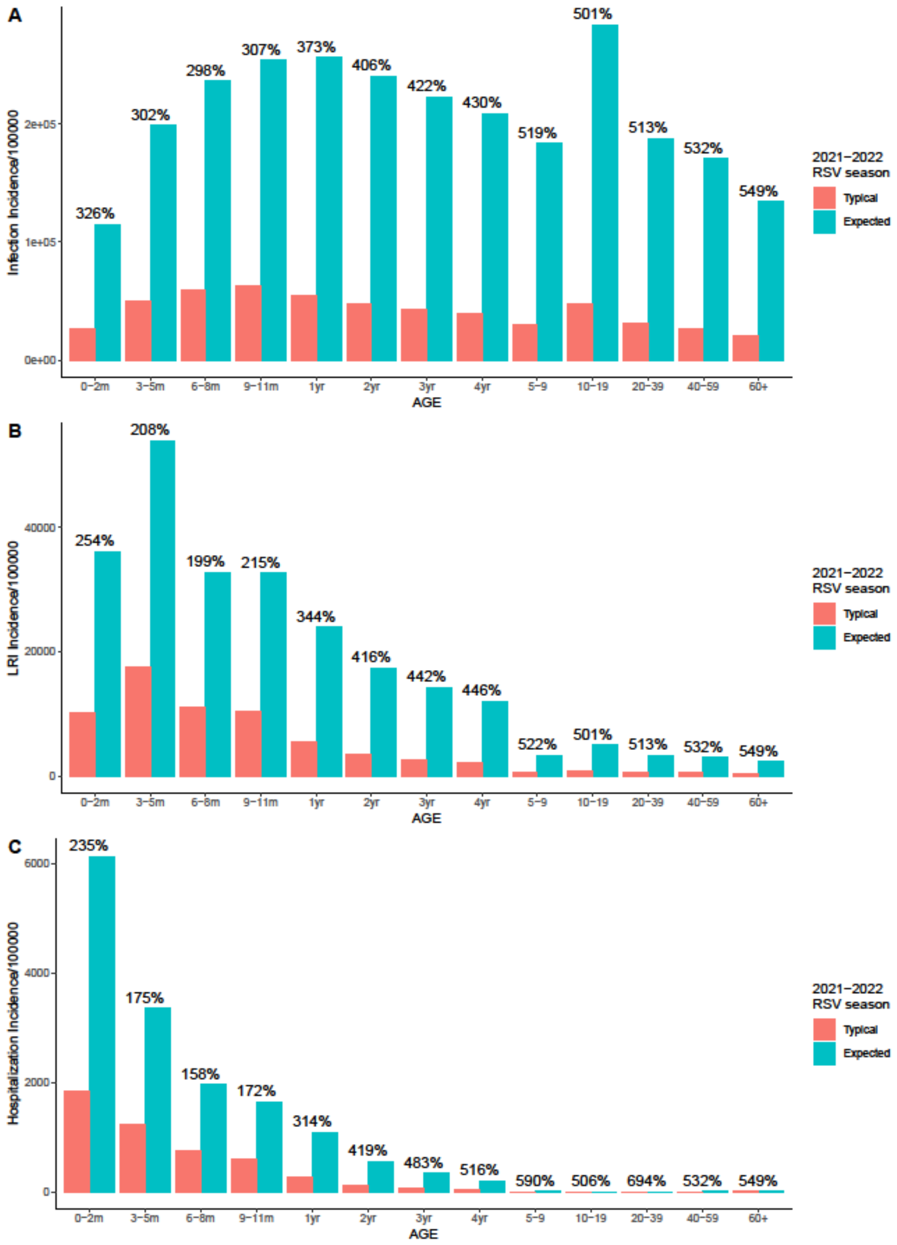

eFigure 18. Age distribution of RSV infections, lower respiratory infections (LRIs) and hospitalizations under the assumption that biennial epidemics are greater in odd years, 2021-2022 RSV season. Panel A to C correspond to RSV infections, RSV LRIs and RSV hospitalizations. The red color bars show the counterfactual incidence of RSV cases during 2021 to 2022 RSV season if there was no COVID-19 pandemic and no mitigation measures in place. The blue color bars show the expected incidence of RSV cases under Scenario 2 during the 2021-2022 RSV season. The numbers on the top show the percentage difference between the expected incidence and the counterfactual incidence in each age group.

© 2021 Zheng Z et al. *JAMA Network Open.*

eReferences

1. Prem K, Cook AR, Jit M. Projecting social contact matrices in 152 countries using contact surveys and demographic data. *PLoS Comput Biol.* 2017;13(9):e1005697.
2. van Hoek AJ, Andrews N, Campbell H, Amirthalingam G, Edmunds WJ, Miller E. The social life of infants in the context of infectious disease transmission; social contacts and mixing patterns of the very young. *PloS one.* 2013;8(10):e76180.
3. Wallinga J, Teunis P, Kretzschmar M. Using data on social contacts to estimate age-specific transmission parameters for respiratory-spread infectious agents. *American journal of epidemiology.* 2006;164(10):936-944.
4. Glezen WP, Taber LH, Frank AL, Kasel JA. Risk of Primary Infection and Reinfection With Respiratory Syncytial Virus. *American Journal of Diseases of Children.* 1986;140(6):543-546.
5. Ohuma EO, Okiro EA, Ochola R, et al. The natural history of respiratory syncytial virus in a birth cohort: the influence of age and previous infection on reinfection and disease. *American journal of epidemiology.* 2012;176(9):794-802.
6. Hacimustafaoglu M, Celebi S, Aynaci E, et al. The progression of maternal RSV antibodies in the offspring. *Archives of disease in childhood.* 2004;89(1):52-53.
7. Jans J, Wicht O, Widjaja I, et al. Characteristics of RSV-Specific Maternal Antibodies in Plasma of Hospitalized, Acute RSV Patients under Three Months of Age. *PloS one.* 2017;12(1):e0170877.
8. Henderson FW, Collier AM, Clyde WA, Jr., Denny FW. Respiratory-syncytial-virus infections, reinfections and immunity. A prospective, longitudinal study in young children. *The New England journal of medicine.* 1979;300(10):530-534.
9. Hall CB, Geiman JM, Biggar R, Kotok DI, Hogan PM, Douglas GR, Jr. Respiratory syncytial virus infections within families. *The New England journal of medicine.* 1976;294(8):414-419.
10. Nokes DJ, Okiro EA, Ngama M, et al. Respiratory syncytial virus infection and disease in infants and young children observed from birth in Kilifi District, Kenya. *Clinical infectious diseases : an official publication of the Infectious Diseases Society of America.* 2008;46(1):50-57.
11. Wright PF, Gruber WC, Peters M, et al. Illness severity, viral shedding, and antibody responses in infants hospitalized with bronchiolitis caused by respiratory syncytial virus. *The Journal of infectious diseases.* 2002;185(8):1011-1018.
12. Walsh EE, Peterson DR, Kalkanoglu AE, Lee FE, Falsey AR. Viral shedding and immune responses to respiratory syncytial virus infection in older adults. *The Journal of infectious diseases.* 2013;207(9):1424-1432.
13. Takeyama A, Hashimoto K, Sato M, Kawashima R, Kawasaki Y, Hosoya M. Respiratory syncytial virus shedding by children hospitalized with lower respiratory tract infection. *Journal of medical virology.* 2016;88(6):938-946.
14. Munywoki PK, Koech DC, Agoti CN, et al. Influence of age, severity of infection, and co-infection on the duration of respiratory syncytial virus (RSV) shedding. *Epidemiology and infection.* 2015;143(4):804-812.
15. Pitzer VE, Lipsitch M. Exploring the relationship between incidence and the average age of infection during seasonal epidemics. *J Theor Biol.* 2009;260(2):175-185.
16. Menzies NA, Soeteman DI, Pandya A, Kim JJ. Bayesian Methods for Calibrating Health Policy Models: A Tutorial. *Pharmacoeconomics.* 2017;35(6):613-624.
17. Alkema L, Raftery AE, Brown T. Bayesian melding for estimating uncertainty in national HIV prevalence estimates. *Sex Transm Infect.* 2008;84 Suppl 1:i11-i16.
18. contributors W. Maximum a posteriori estimation. https://en.wikipedia.org/w/index.php?title=Maximum_a_posteriori_estimation&oldid=932761908. Accessed 30 March 2020 03:24 UTC.

© 2021 Zheng Z et al. *JAMA Network Open.*

19. Ochola R, Sande C, Fegan G, et al. The level and duration of RSV-specific maternal IgG in infants in Kilifi Kenya. *PloS one.* 2009;4(12):e8088.
20. Hall CB, Douglas RG, Jr., Geiman JM. Respiratory syncytial virus infections in infants: quantitation and duration of shedding. *The Journal of pediatrics.* 1976;89(1):11-15.
21. Monto AS, Bryan ER, Rhodes LM. The Tecumseh study of respiratory illness. VII. Further observations on the occurrence of respiratory syncytial virus and Mycoplasma pneumoniae infections. *American journal of epidemiology.* 1974;100(6):458-468.
22. Pitzer VE, Viboud C, Alonso WJ, et al. Environmental Drivers of the Spatiotemporal Dynamics of Respiratory Syncytial Virus in the United States. *PLOS Pathogens.* 2015;11(1):e1004591.
23. Munywoki PK, Koech DC, Agoti CN, et al. Frequent Asymptomatic Respiratory Syncytial Virus Infections During an Epidemic in a Rural Kenyan Household Cohort. *The Journal of infectious diseases.* 2015;212(11):1711-1718.
24. Hall CB, Long CE, Schnabel KC. Respiratory syncytial virus infections in previously healthy working adults. *Clinical infectious diseases : an official publication of the Infectious Diseases Society of America.* 2001;33(6):792-796.
25. Tong S, Amand C, Kieffer A, Kyaw MH. Incidence of respiratory syncytial virus related health care utilization in the United States. *J Glob Health.* 2020;10(2):020422.
26. Takashima MD, Grimwood K, Sly PD, et al. Epidemiology of respiratory syncytial virus in a community birth cohort of infants in the first 2 years of life. *European journal of pediatrics.* 2021.
27. Fisher RG, Gruber WC, Edwards KM, et al. Twenty years of outpatient respiratory syncytial virus infection: a framework for vaccine efficacy trials. *Pediatrics.* 1997;99(2):E7.
28. Stranak Z, Saliba E, Kosma P, et al. Predictors of RSV LRTI Hospitalization in Infants Born at 33 to 35 Weeks Gestational Age: A Large Multinational Study (PONI). *PloS one.* 2016;11(6):e0157446.
29. Baker RE, Park SW, Yang W, Vecchi GA, Metcalf CJE, Grenfell BT. The impact of COVID-19 nonpharmaceutical interventions on the future dynamics of endemic infections. *Proc Natl Acad Sci U S A.* 2020;117(48):30547-30553.
30. Feehan DM, Mahmud AS. Quantifying population contact patterns in the United States during the COVID-19 pandemic. *Nat Commun.* 2021;12(1):893.
31. Yan Y, Malik AA, Bayham J, Fenichel EP, Couzens C, Omer SB. Measuring voluntary and policy-induced social distancing behavior during the COVID-19 pandemic. *Proc Natl Acad Sci U S A.* 2021;118(16).
32. Air Travel - Total - Seasonally Adjusted. U.S. Department of Transportation. Bureau of Transportation Statistics. February 2021; https://data.bts.gov/Research-and-Statistics/Air-Travel-Total-Seasonally-Adjusted/gmwv-css9. Accessed May 5, 2021.
33. RSV surveillance activity summary. Florida Department of Health. May 16, 2021; http://www.floridahealth.gov/diseases-and-conditions/respiratory-syncytial-virus
